# Development of a cell-penetrating peptide for intracellular delivery of a biologically active HPV E6 inhibitor in cervical cancer cells

**DOI:** 10.1186/s13046-026-03805-4

**Published:** 2026-08-12

**Authors:** Luisa Dassi, Anna Lucia Tornesello, Marian Vincenzi, Marilisa Leone, Vincenzo Ingangi, Wuyuan Lu, Andrea Cerasuolo, Tiziana Pecchillo Cimmino, Sara Amiranda, Mariapia Napolitano, Palma Tirino, Franca Maria Tuccillo, Luigi Buonaguro, Vincenza De Gregorio, Giorgia Imparato, Franco Maria Buonaguro, Maria Lina Tornesello

**Affiliations:** 1 Molecular Biology and Viral Oncology Unit, Istituto Nazionale Tumori IRCCS Fondazione G. Pascale, Napoli, Italy; 2 Innovative Immunological Models Unit, Istituto Nazionale Tumori IRCCS Fondazione G. Pascale, Napoli, Italy; 3 Institute of Biostructures and Bioimaging, National Research Council of Italy (IBB-CNR), Napoli, Italy; 4 Preclinical Models of Tumor Progression Unit, Istituto Nazionale Tumori IRCCS Fondazione G. Pascale, Napoli, Italy; 5 Shanghai Institute of Infectious Disease and Biosecurity, School of Basic Medical Sciences, Shanghai Medical College, Fudan University, Shanghai, China; 6 Department of Biology, University of Naples “Federico II”, Via Cinthia, Napoli, Italy; 7 Center for Advanced Biomaterials for Healthcare, Istituto Italiano Di Tecnologia-IIT, Napoli, Italy

**Keywords:** Human papillomavirus type 16 E6, HPV16 E6, HPV 18 E6, cell penetrating peptide, CPP, Cervical carcinoma, Oncosuppressor p53, Ubiquitination

## Abstract

**Background:**

The E6 oncoprotein of high-risk human papillomaviruses (HPVs) promotes cervical carcinogenesis by forming a complex with the ubiquitin ligase E6AP to mediate p53 proteasomal degradation. A 15-amino-acid peptide (pep11) was shown to disrupt the HPV16 E6/E6AP complex, to restore p53 stability and to induce apoptosis in HPV16-positive cancer cells. In this study, a short cell-penetrating peptide (CPP) was conjugated to the pep11 N-terminus to improve its solubility and intracellular delivery in HPV-positive cancer cells.

**Methods:**

CPP-pep11 was synthesized using Boc chemistry. The interactions of CPP-pep11 with HPV16 and HPV18 E6 were modelled using AlphaFold2, while peptide structures were characterised by NMR spectroscopy. HPV16-positive SiHa and HPV18-positive C4-I cervical cancer cells were treated with CPP-pep11 (0.5–20 µM) for 24–72 h. Effects on proliferation and clonogenicity were assessed using xCELLigence and colony formation assays. Peptide uptake and localization were analysed by confocal microscopy and differential cell fractionation, while p53 expression and viability were evaluated by Western blotting and cytotoxicity assays.

**Results:**

AlphaFold2 modelling suggested that CPP-pep11 can interact with both HPV16 E6 and HPV18 E6, with a more stable predicted binding to HPV16 E6. CPP-pep11 was soluble in water at 20 µM and, according to NMR analysis, predominantly adopted a disordered conformation. CPP-pep11 efficiently entered the SiHa and C4-I cell membranes, mainly localised to the cytoplasm at lower concentrations (0.5–5 µM) and to the nucleus at higher concentrations (10–20 µM). CPP-pep11 induced dose- and time-dependent inhibition of proliferation, with a significant reduction in colony formation in both cell lines. Treatment also restored p53 levels at 48 and 72 h, consistent with disruption of E6-mediated degradation.

**Conclusions:**

In conclusion, CPP-pep11 is a novel peptide-based inhibitor of HPV16 E6 and HPV18 E6 with therapeutic potential in HPV-associated malignancies by restoring p53 levels and inhibiting cancer cell proliferation.

**Supplementary Information:**

The online version contains supplementary material available at https://doi.org/10.1186/s13046-026-03805-4.

## Introduction

Cervical cancer continues to represent a major global health problem, ranking as the fourth most common malignancy among women worldwide in both incidence and mortality, with an estimated 660,000 new cases and 350,000 deaths reported in 2022 [1, 2]. Persistent infection with high risk HPVs is a well-established etiological cause of cervical cancer [3]. The oncogenic potential varies among diverse viral genotypes, with HPV 16 and 18 carrying a particularly high risk of progression from intraepithelial lesions to invasive cervical cancer [4]. Worldwide, the distribution of the most prevalent carcinogenic genotypes, namely HPV 16, 18, 31, 33, 35, 45, 52 and 58, is generally consistent across diverse geographic regions [5]. Collectively, these genotypes account for approximately 91% of cervical cancers, with HPV16 and HPV18 alone responsible for about 71% of cases [6].

A central event in cervical carcinogenesis is the integration of HPV DNA into the host genome, which frequently causes disruption of the E1/E2 open reading frames and the loss of function of the viral transcription repressor E2 [7]. The resulting activation of the HPV early promoter drives sustained expression of E6 and E7 viral oncoproteins in the cervical epithelium, which are essential for both the initiation and maintenance of the malignant phenotype [8]. These oncoproteins drive carcinogenesis by inactivating p53 and retinoblastoma protein (pRb) tumour suppressors, respectively, as well as many other cellular proteins, causing uncontrolled cell proliferation and genome instability [9–12].

The HPV16 E6 protein is a 151-amino-acid oncoprotein composed of two zinc-binding domains linked by an α-helix. Crystallographic studies have shown that binding of the E6AP LxxLL motif induces the formation of a hydrophobic pocket at the interface of the two domains, enabling E6 to recruit p53 for ubiquitin-mediated proteasomal degradation [13]. Recently, new interaction sites between E6AP and HPV16 E6 have been identified through the analysis of the E6AP/E6/p53 complex performed by cryo-electron microscopy. They include a novel N-terminal ordered domain in E6AP (residues 236–515) terminating in a long alpha-helix that directly interacts with the linker helix comprised between the two zinc-binding domains in E6. Interestingly, this assembly displaces the E2 ligase UbcH7 from the cognate E6AP carboxyl terminus domain, suggesting a mechanism of allosteric regulation. Combined with in *vitro* ubiquitination and yeast two-hybrid assays, this 3D reconstruction provided new insights into the conformational dynamics driving p53 ubiquitination [14].

The E6/E6AP-mediated p53 ubiquitination and proteasomal degradation causes disruption of cell cycle regulation and inhibition of apoptosis, thus promoting genomic instability and progression of cervical carcinogenesis [15]. Importantly, HPV-positive cervical carcinomas and their derived cell lines generally retain wild-type p53, which is functionally inactivated by the E6 oncoprotein, making disruption of this interaction a promising therapeutic strategy [16]. Previous approaches have focused on preventing p53 interactions with its inhibitors or reactivating the TP53 transcriptional activity by blocking MDM2-mediated degradation [17]. More recently, new inhibitors of E6 protein have been identified which are able to disrupt the E6-E6AP interaction and to prevent p53 degradation in HPV-positive cells [17–19]. However, the absence of a well-defined ligand-binding pocket in E6 renders the viral protein a challenging target for small molecules [20, 21]. Conversely, peptide-based inhibitors, particularly those mimicking the LxxLL motif of E6AP, are better suited for targeting E6 interactions. These peptides competitively inhibit E6-E6AP binding and restore p53 levels when introduced in HPV-positive cells [22–24]. Dymalla et al. identified novel peptides able to inhibit HPV E6, which presented a new binding motif (CxChxCh) in addition to the canonical LxxLL motif [23]. Among them, a new peptide carrying only the CxChxCh motif, namely pep11, showed selective binding to HPV16 E6 with higher affinity than E6AP peptide, which was shown to inhibit the growth of HPV16-positive cells and to restore p53 levels [25]. However, the chemical synthesis of pep11 was challenging due to its intrinsic hydrophobicity, resulting in low yields and poor solubility in aqueous media.

Cell-penetrating peptides are short peptides capable of crossing cellular membranes, which are classified according to their origin as protein-derived, chimeric, or synthetic CPPs [26]. Most of them are linear, L-amino-acid-based sequences produced via chemical synthesis, and categorized by physicochemical properties into amphipathic, hydrophobic and cationic types [27]. The majority of cationic CPPs derives from Tat and Penetratin natural peptides and usually contains more than five positively charged amino acids, with cationic arginine- and tryptophan-rich sequences showing the highest cellular uptake [28]. In particular, arginine-rich peptides have the ability to bind the phosphate groups and to induce a strong distortion in the phospholipid bilayer, thus facilitating their translocation through membranes [29].

In the present study, we designed and developed a cell-penetrating peptide by linking a Tat-derived sequence, consisting of six arginine residues, to pep11 (CPP-pep11) and characterized its conformation. We modelled the structure of HPV16 E6 and HPV18 E6 and their possible interactions with CPP-pep11 by using AlphaFold2. Next, we evaluated CPP-pep11 cellular uptake mechanism, subcellular localization and stability as well as the anti-proliferative effects on HPV16- and HPV18-positive cervical cancer cells and its ability to prevent p53 degradation.

## Materials and methods

### Peptide synthesis

The EFGSGCSCIVCIGLI-NH_2_ (pep11) precursor peptide, RRRRRR-EFGSGCSCIVCIGLI-NH_2_ (CPP-pep11) peptide and RRRRRR-CGVSICGLSFEICIG-NH_2_ (CPP-SCRpep11) scrambled peptide were synthesized using an automated peptide synthesizer (Applied Biosystems, Foster City, CA, USA). Peptide synthesis was carried out on a 0.25 mmol scale using N, N-Diisopropylethylamine (DIPEA) for in situ neutralization in combination with 2-(1 H-benzotriazol-1-yl)-1,1,3,3-tetramethyluronium hexafluorophosphate (HBTU) activation, following the tert-butyloxycarbonyl (Boc) solid-phase peptide synthesis [30]. 4-Methylbenzhydrylamine (MBHA) resin with a loading of 0.72 mmol/g served as the solid support. Double couplings were performed using Boc-amino acids (1.2 mmol) activated for 3 min in N, N-Dimethylformamide (DMF) with 1 mmol HBTU in the presence of 20% DIPEA (v/v) and coupled gradually over 10 min. N-Boc protecting groups were removed using absolute trifluoroacetic acid (TFA), while DMF and dichloromethane (DCM) were used for washing cycles. After assembly of the peptide sequences on the solid support and removal of the Boc group from the N-terminal residue, 1.2 equivalents of Biotin N-hydroxysuccinimide ester dissolved in DMF, along with 2 equivalents of DIPEA, were added to half of the resin for both CPP-pep11 and CPP-SCRpep11. Coupling reactions were carried out overnight at room temperature with continuous stirring. Coupling efficiency was monitored with Kaiser’s test. All peptides’ derivatives were treated for the cleavage with anhydrous hydrofluoric acid (HF) in the presence of 5% p-cresol at 0 °C for 1 h, followed by precipitation with cold diethyl ether. The side chain protections included: Arg(Tosyl), Cys(4MeBzl), Glu(OcHxl) and Ser(Bzl). Amino acids and resin were purchased from Bachem Americas Inc. (Torrance, CA, USA). TFA was obtained from Halocarbon, LLC (Peachtree Corners, GA, USA), methanol and DCM were supplied by Fisher Scientific (Pittsburgh, PA, USA), DIPEA from Fluka Chemical Corp. (part of Merck Group, Darmstadt, Germany) and HBTU solution was purchased from Oakwud Chemical (Estill, SC, USA). NHS-Biotin was purchased from Millipore Sigma (Burlington, MA, USA). All solvents were of reagent grade and used without further purification.

The ovalbumin-derived cell penetrating peptide Biot-RRRRRR-SIINFEKL was synthesized through solid phase strategy in continuous flow with automatic synthesizer Syro I (Biotage, Sweden) using the 9-fluorenylmethoxycarbonyl (Fmoc) chemistry. The 2-chlorotrityl chloride resin (Iris Biotech GmbH) was preloaded with Fmoc-Leu-OH (Sigma-Aldrich), reaching a final loading of 0.15 mmol/g. The synthesis was performed on a 0.025 mmol scale using as protected amino acids Fmoc-Lys(Boc)-OH, Fmoc-Glu(OtBu)-OH, Fmoc-Phe-OH, Fmoc-Asn(Trt)-OH, Fmoc-Ile-OH and Fmoc-Ser(tBu)-OH, purchased from Iris Biotech GmbH, and Fmoc-Arg(Pbf)-OH purchased from Merck. The automatic synthesis proceeded through Fmoc deprotection steps using the 40% piperidine in DMF solution and Fmoc-amino acid single coupling steps using 4 mmol Fmoc-amino acid and HBTU and 8 mmol of DIPEA. Peptides were treated for the cleavage with TFA in the presence of 5% Triisopropylsilane (TIS) and 5% H_2_O at 0 °C for 3 h, followed by precipitation with cold diethyl ether. Crude peptides were purified by reverse-phase high-performance liquid chromatography (RP-HPLC) on a Waters C18 column, eluted with H₂O/0.1% TFA (A) and CH₃CN/0.1% TFA (B) using a linear gradient from 20 to 40% B over 2 h at 40 mL/min. Molecular weight was confirmed by electrospray ionization mass spectrometry (ESI-MS).

### NMR spectroscopy analysis

NMR spectra of CPP-pep11 were acquired on a Varian Unity spectrometer operating at a proton frequency of 500 MHz, situated at the DiSTABif (Department of Environmental, Biological and Pharmaceutical, Science and Technologies), of the University of Campania Luigi Vanvitelli (Caserta, Italy). NMR studies were performed at 298 K for the four pep11 variants: CPP-pep11; Biot-CPP-pep11; CPP-SCRpep11; Biot-CPP-SCRpep11. NMR samples were prepared by dissolving weighted amounts of peptide powders into sample buffer consisting of H_2_O: D_2_O (98% D, Sigma-Aldrich, Merck KGaA, Darmstadt, Germania) 90:10 v/v supported also with 8 mM DL-1,4-Dithiothreitol (DTT-D₁₀, 98% D, Cambridge Isotope Laboratories, Inc. provided by Spectra 2000 Srl, Rome, Italy). NMR analyses were performed using total sample volumes of 600 µL (Supplementary Tables S1-S4).

The following experiments were performed for each peptide sample: 1D [^1^H]; 2D [^1^H, ^1^H] Total Correlation Spectroscopy (TOCSY) [31] (70 ms mixing time), Nuclear Overhauser Enhancement Spectroscopy (NOESY) [32] (300 ms mixing times), 2D [^1^H, ^1^H] Rotating Frame Overhauser Enhancement Spectroscopy (ROESY) (250 ms mixing time) [33] and Double Quantum-Filtered Correlated Spectroscopy (DQFCOSY) [34]. Typical parameter set for 2D spectra included 64 transients; 4 K data points and 256 increments. Suppression of the water signal was achieved through the Double Pulsed Field Gradient Spin Echo (DPFGSE) pulse scheme [35]. Chemical shifts were referenced to Trimethylsilyl-3-propionic acid sodium salt-D4, (TSP 99% D, Armar Scientific, Switzerland) (0.0 ppm) that was added to each NMR sample. Software used to process and analyse NMR spectra included VNMRJ (V. 2.2) (Varian Inc./Agilent Technologies, Milan, Italy) and NEASY [36] contained in CARA (Computer Aided Resonance Assignment) (http://cara.nmr.ch/doku.php/). The ^1^H chemical shift assignments were achieved by a well-established protocol relying mostly on the comparison of TOCSY and NOESY spectra [37]. Differences between observed ^1^Hα chemical shift and random coil (r.c.) values (i.e., Δδ(Hα) = δ(Hα)observed – δ(Ηα)r.c.) were evaluated by employing the strategy proposed by Kjaergaard and collaborators for estimating random coil chemical shift values (https://www1.bio.ku.dk/english/research/bms/sbinlab/randomchemicalshifts1, Access date 19/12/25) [38]. Schematic representations of short and medium range NOEs were obtained with the software CYANA (v. 2.1) and for this purpose peak volumes in NOESY spectra were manually integrated and then converted into upper – limits interproton distances with the routine “CALIBA” of CYANA [39, 40].

### AlphaFold2 models of the HPV16 E6/CPP-pep11 and HPV18 E6/CPP-pep11 peptide complexes

AlphaFold2 [41, 42], accessed in the ColabFold server [43] (https://colab.research.google.com/github/sokrypton/ColabFold/blob/main/AlphaFold2.ipynb#scrollTo=kOblAo-xetgx, accessed on 17 April 2026) was exploited to obtain the models of the HPV16 E6/CPP-pep11 and HPV18 E6/CPP-pep11 peptide complexes starting from the sequences of either the HPV16 E6 protein (P03126), or the HPV18 E6 protein (P06463) and the CPP-pep11 peptide. Different parameters were used to run the AlphaFold2 predictions. Specifically, the “PDB100” option was chosen for the “template mode” value and thus the runs were achieved considering homologous structure templates; “alphafold2_multimer_v3”, 48, 0.0, 0 (i.e., unlimited), “complete”, “512:1024” and “1” values were established for “model type”, “num_recycles”, “recycle_early_stop_tolerance”, “relax_max_iterations”, “pairing strategy”, “max_msa” and “num_seeds” parameters, respectively. Default values were maintained for the other parameters [43]. Five output structures were produced and to each of them, post-prediction relaxation via gradient descent in the Amber force field was applied [44, 45]. The five models of each complex were ranked based on the pTM and ipTM values following the formula 0.2 × pTM + 0.8 × ipTM [43].

### Diagrams of intermolecular contacts by LigPlot+

2D diagrams of intermolecular interactions characterizing the five AlphaFold2 predicted structures of the HPV16 E6/CPP-pep11 and HPV18 E6/CPP-pep11 complexes were generated with LigPlot+ (version 2.2.8) [46]. H-bonds were identified using the following thresholds: 2.5 Å (H-acceptor distance) and 3.35 Å (donor-acceptor distance). Regarding non-bonded protein-peptide interactions, these were selected by choosing cut-off values equal to 2.9 Å and 3.9 Å for the minimum and maximum distances between any atoms in any residue, respectively [46, 47]. Salt bridges between negatively and positively charged residues were assessed as previously reported [48].

### Cell culture conditions and CPP peptide treatment

The human cervical cancer cell lines SiHa (ATCC^®^ HTB-35™) and C4-I (ATCC^®^ CRL-1594™) were obtained from the American Type Culture Collection (ATCC, Manassas, VA, USA). SiHa and C4-I cells contain integrated HPV16 and HPV18 DNA, respectively. SiHa cells were cultured in high-glucose Dulbecco’s Modified Eagle Medium (DMEM), while C4-I cells were maintained in RPMI-1640 medium. Both media were supplemented with 10% foetal bovine serum (FBS), 2 mM L-glutamine, and 100 U/mL penicillin plus 100 µg/mL streptomycin. Cells were incubated at 37 °C in a humidified atmosphere containing 5% CO₂.

CPP peptides were dissolved in ultrapure water (Milli-Q system) and their concentrations were determined spectrophotometrically using a NanoDrop 2000c instrument (Thermo Fisher Scientific, Waltham, MA, USA). Cells were seeded and allowed to adhere for 24 h prior to treatment with increasing final concentrations of CPP peptides (0.5, 2, 5, 10, and 20 µM). For cell viability and cytotoxicity assays, non-biotinylated CPP peptides were used. Before peptide treatment, the culture medium was removed and replaced with serum-free medium containing the indicated concentrations of CPP peptides. Cells were incubated for 6 h at 37 °C, then supplied with fresh complete growth medium and maintained in culture for an additional 72 h.

### Confocal microscopy analysis

Cells were treated with biotinylated CPP peptides and analysed in confocal microscopy for their subcellular localization. SiHa cells and C4-I (2 × 10^4^/sample) were seeded onto glass-bottom 8-well µ-Slides (ibidi GmbH, Martinsried, Germany) and allowed to adhere for 24 h prior to treatment with biotinylated CPP-peptides (0, 0.5, 2, 5, 10, and 20 µM). Following treatment, cells were fixed with 4% paraformaldehyde (PFA) for 15 min at room temperature and permeabilized with 1× PBS containing 0.1% Triton X-100 for 10 min. Cells were blocked in blocking buffer (1× PBS, 0.05% Triton X-100, 5% FBS) and incubated overnight with Streptavidin-Alexa Fluor 488 (Invitrogen, Carlsbad, CA, USA; dilution 1:200) for biotinylated peptides detection. For microtubule visualization, cells were incubated with an anti-α-tubulin primary antibody (Cell Signaling Technology, Danvers, MA, USA; dilution 1:100), followed by the appropriate fluorophore-conjugated secondary antibody. In addition, cells were incubated with Phalloidin-Alexa Fluor 647 for F-actin staining according to the manufacturer’s instructions (Invitrogen; dilution 1:400). Nuclei were counterstained with 4 µM Hoechst 33342 (Thermo Fisher Scientific, Waltham, MA, USA). Images were acquired using a Leica Stellaris 5 DMI8 confocal microscope (Leica Microsystems, Wetzlar, Germany) equipped with a 63X oil-immersion objective. The list of antibodies used in this study is provided in Supplementary Table S5.

### Western blotting analysis

Cells were seeded in 6-well plates (3 × 10⁵ cells/well) and treated with CPP peptides at final concentrations ranging from 0.5 µM to 20 µM for 24, 48, and 72 h to monitor peptide stability over time and variations in p53 protein levels. After treatment, cells were rinsed three times with 1× PBS and scraped into 300 µL RIPA buffer (50 mM Tris-HCl, 150 mM NaCl, 1.0% [v/v] NP-40, 0.5% [w/v] sodium deoxycholate, 1.0 mM EDTA, 0.1% [w/v] SDS, and 0.01% [w/v] sodium azide, pH 7.4) supplemented with 2 mM PMSF (200 mM), 1 mM sodium orthovanadate (100 mM), and 1.5% [v/v] protease inhibitor cocktail (Santa Cruz Biotechnology, Dallas, TX, USA; sc-24948). Lysates were incubated on ice for 30 min and centrifuged at 18,000 RCF for 20 min at 4 °C. Supernatants were stored at -80 °C. Protein concentrations were determined using the Bradford method.

Fifteen micrograms of proteins were separated by electrophoresis using 4–15% Mini-PROTEAN^®^ TGX™ Precast Protein Gels (Bio-Rad, Hercules, CA, USA) for p53 detection. CPP-pep11 was analysed using 16% Tricine-SDS-PAGE gels as previously described [49]. Proteins were transferred to PVDF membranes (Thermo Fisher Scientific) in transfer buffer containing 192 mM glycine, 25 mM Tris-HCl (pH 8.0), and 20% methanol (v/v) at 90 V for 90 min.

Membranes were blocked in 5% [w/v] non-fat dry milk (Santa Cruz Biotechnology, sc-2324) in Tris-buffered saline (TBS; 20 mM Tris-HCl, 140 mM NaCl, pH 7.5) and incubated with primary antibodies: p53 monoclonal antibody (Santa Cruz Biotechnology; DO-1, sc-126; 1:500 dilution) and anti-actin monoclonal antibody (Merck; MAB1501; 1:1000 dilution) in TBST (TBS + 0.1% Tween-20) containing 1% milk. Membranes were washed three times with TBST and incubated for 2 h at room temperature with secondary antibodies: goat anti-mouse IgG-Horseradish peroxidase (IgG-HRP) (Santa Cruz Biotechnology; sc-2302; 1:2000 or 1:5000 dilution) in TBST with 1% milk, or streptavidin-HRP conjugate (Invitrogen; SA100-01; 1:5000 dilution) in TBST with 1% milk to detect biotinylated peptides. After the final washes, protein bands were visualized using the iBright™ CL1500 Imaging System (Thermo Fisher Scientific).

### Real-time monitoring of cell proliferation with xCELLigence

Cell proliferation was monitored using E-16-well plates and the xCELLigence real-time cell analysis (RTCA) system (Acea Bioscience, a Roche Diagnostics company, Basel, Switzerland). The instrument was used to measure impedance changes across interdigitated gold microelectrodes on the well bottom, correlating with cell attachment, spreading, and proliferation, and such parameters expressed as cell index. Cells (2.5 × 10³ cells/well) were seeded and allowed to adhere for 24 h before treatment. Cells were incubated with CPP peptides for six hours and then supplemented with growth medium containing 10% FBS. Cytotoxicity and effects on proliferation were assessed over 72 h using CPP concentrations ranging from 0 to 20 µM. Doubling times were calculated during the exponential growth phase. All analyses were performed in at least two independent experiments, each performed in quadruplicate wells. Data from the xCELLigence RTCA system were analysed using normalized cell index plots.

### Cell viability assay

Cell viability was assessed using the Trypan Blue exclusion method. Cells (5 × 10⁴ cells/well) were seeded in 24-well plates 24 h prior to treatment. CPP peptides were added at varying concentrations, and cell numbers were determined at 24, 48, and 72 h using a Bürker counting chamber under a light microscope. Cell viability was assessed by staining with 0.4% Trypan Blue (Thermo Fisher Scientific). Experiments were performed in duplicate for each condition and repeated at least three times.

### Clonogenic assay for cell survival

SiHa (800 cells/well) or C4-I (1000 cells/well) cells were plated in 6-well plates and allowed to grow until the formation of 3–4 cells per colony. Cells were then treated with CPP peptides at the indicated concentrations. Culture medium containing CPPs was refreshed after 72 h, and cells were further cultured for an additional 96 h, during which the effect on cell proliferation became evident. After a total culture period of approximately 12 days, cells were fixed with 4% formalin (Thermo Fisher Scientific) and stained with 0.1% crystal violet (Sigma-Aldrich). Plates were scanned using a ZEISS Axiovert 25 microscope (Zeiss) and images were captured with the ZEN 2.3 Lite software.

## Results

### Design and development of the CPP-pep11 interference peptide

The peptide pep11 has been previously shown to specifically bind HPV16 E6, thereby preventing the recruitment of p53 for proteasome-mediated degradation [23]. To improve pep11 solubility while preserving its E6-binding capability, the key residues responsible for the interaction were identified using alanine-scanning mutagenesis. This analysis revealed that the hydrophobic C-terminal region is critical for binding, whereas substitutions at the N-terminus outside the consensus motif are largely tolerated. Based on these findings, optimized variants pep11* and pep11** were designed, both retaining strong binding affinity for HPV16 E6 and weaker binding affinity for HPV18 E6 [23].

In this study, we synthesized the native pep11 peptide and enhanced its production yield, solubility, and intracellular delivery by conjugating it to a cell-penetrating peptide (CPP). To achieve this, a polyarginine (RRRRRR) sequence was covalently linked to the N-terminal end of pep11, generating CPP-pep11. This modification enhanced peptide solubility, allowing preparation of stable solutions with a concentration of up to 20 µM in water. A scrambled version of the CPP-pep11 sequence (CPP-SCRpep11) was generated by randomly rearranging the amino acids of the original pep11 and used as a control. Furthermore, the CPP moiety was conjugated to the SIINFEKL ovalbumin-derived sequence (CPP-OVA_257−264_) to be used as additional control. The CPP-pep11, CPP-SCRpep11 and CPP-OVA_257−264_ were biotinylated to evaluate cellular uptake and subcellular localization.

The CPP-pep11 and CPP-SCRpep11 peptides were synthesized using the BOC strategy, in which N-terminal amino groups are protected with t-butyloxycarbonyl groups during stepwise solid-phase assembly, followed by cleavage from the resin. The CPP-OVA_257−264_ peptide was synthesized by conventional Fmoc solid phase synthesis. Peptides were purified by RP-HPLC and characterized by LC-ESI-MS. The experimental M/Z values for multiple charge states (Z = 3 and Z = 4) closely matched the theoretical masses for peptides CPP-pep11, Biot-CPP-pep11, CPP-SCRpep11, and Biot-CPP-SCRpep11, CPP-OVA_257−264_ and Biot-CPP-OVA_257−264_ confirming successful synthesis (Table 1).

**Table 1:** Physicochemical and analytical characterization of the peptides used in this study. For each peptide, the amino-acid sequence, theoretical molecular weight, and experimentally determined MW values obtained by mass spectrometry are reported

Peptide name	Sequence	Theoretical MW* (g/mol)			Experimental MW* (g/mol)		RP-HPLC*	
		M/Z (g/mol)	M/z Z = 3	M/z Z = 4	M/z Z = 3	M/z Z = 4	t_*R*_	Purity (%)
pep11	EFGSGCSCIVCIGLI-NH_2_	1499.8	499.9	374.9	**n.a.**	**n.a.**	**n.a.**	**n.a.**
CPP-pep11	RRRRRR-EFGSGCSCIVCIGLI-NH_2_	2436.9	812.3	609.2	813.25	610.2	18.13	> 90%
Biot-CPP-pep11	Biot-RRRRRR-EFGSGCSCIVCIGLI-NH_2_	2663.0	887.6	665.7	888.43	666.6	18.16	> 90%
SCRpep11	CGVSICGLSFEICIG-NH_2_	1499.8	499.9	374.9	**n.a.**	**n.a.**	**n.a.**	**n.a.**
CPP-SCRpep11	RRRRRR-CGVSICGLSFEICIG-NH_2_	2436.9	812.3	609.2	813.25	610.2	18.14	> 90%
Biot-CPP-SCRpep11	Biot-RRRRRR-CGVSICGLSFEICIG-NH_2_	2663	887.6	665.7	888.62	666.7	18.17	> 90%
CPP-OVA_257−264_	RRRRRR-SIINFEKL	1900.3	633.4	475.1	634.39	476.0	2.85	> 85%
Biot-CPP-OVA_257−264_	Biot-RRRRRR-SIINFEKL	2125.6	703.5	531.4	709.74	532.6	3.31	> 90%

*Calculated and observed mass-to-charge ratios (m/z) for charge states Z = 3 and Z = 4 are shown, together with reverse-phase HPLC (RP-HPLC) retention time (t_R_) and peptide purity. “n.a.” indicates data not available

The precursor peptides pep11 and SCRpep11 could not be characterized due to their high hydrophobicity, which prevented effective solubilisation. Hydrophobicity predictions using the Bachem Peptide Calculator (Supplementary Figure S1) were validated experimentally. All CPP- and biotin-modified peptides were obtained with > 85% purity, as confirmed by RP-HPLC (Supplementary Figure S2).

To assess the potential for aggregation mediated by the three cysteine residues in the sequences, a stability assay was performed [50]. Peptide CPP-pep11 was dissolved at the concentration of 1 mg/mL in PBS or in dimethyl sulfoxide (DMSO) and stirred in air for up to 72 h. Aggregation was monitored by HPLC at 2, 12, and 72 h. The HPLC profiles of the peptide dissolved in PBS or DMSO and incubated for 72 h were fully superimposable with chromatographic profile of the peptide at time T = 0, indicating no aggregation (Supplementary Figure S3). Mass spectrometry further confirmed the absence of both intermolecular and intramolecular disulphide bond formation.

### NMR studies

NMR structural studies were conducted to get insights into conformational features characterizing the diverse peptides. In particular, both the canonical and scrambled CPP-pep11 peptides, carrying the C-terminal amidation, were analysed in either the N-terminal free or biotinylated forms. NMR analyses were performed in aqueous solution at acidic pH values in the presence of reducing agent dithiothreitol (DTT). Indeed, initial attempts were made to conduct NMR analyses of the Biot-CPP-pep11 under several physiological conditions, such as PBS buffer, but these analyses were hampered by massive precipitation occurring in the NMR tube, likely due to cysteine-mediated sample aggregation through formation of disulphide bridges. The elevated peptide concentrations (i.e., high micromolar-millimolar range) required to get high quality 2D NMR spectra may be responsible for this type of aggregation. In acidic water solutions and in the presence of DTT instead, the cysteines remained in the reduced form and suitable for conducting accurate NMR analyses through 2D[^1^H, ^1^H] NMR spectroscopy (Supplementary Figures S4-S7 and Tables S1-S4). Under the chosen experimental conditions, most ^1^H resonance assignments could be achieved for each peptide (Supplementary Tables S1-S4). However, spectral overlaps were severe in certain peptide regions including the 6 Arginine tails for which it was not possible to unambiguously complete the process of resonance assignments (Supplementary Tables S1-S4).

Secondary structure prediction for the CPP-pep11 and CPP-SCRpep11 was first assessed with the JPred4 webserver, which indicated possible presence of extended conformations covering the segment C14-G19 in CPP-pep11 and V9-C19 in CPP-SCRpep11 (Figure S8) [51]. Next, we carried out analyses of peptide Hα chemical shifts and of Nuclear Overhauser Effects (NOEs) patterns to identify experimentally peptide secondary structure content. Chemical shifts deviations of Hα protons with respect to random coil values are useful NMR parameters to assess the type of secondary structure present in a peptide: positive deviations are indicative of extended β-structuration and negative ones are instead related to helical/turn conformations [38, 52]. As can be clearly seen in the Supplementary Figure S9, both CPP-pep11 and CPP-SCRpep11 in the biotinylated and non-biotinylated forms, present very small positive and negative deviations alternating along the peptide sequence. This kind of scenario does not point out the presence of specific secondary structure elements but indicates instead a more disordered random coil conformation. This observation is further supported by analysis of sequential and medium range NOEs, that are also related to specific secondary structure elements [37] (Supplementary Figure S10). Both CPP-pep11 and CPP-SCRpep11 contain in NOESY spectra just a few sequential NOE contacts that are generally indicative of extended random coil species with no evidence of stable secondary structure [37] (Supplementary Figure S10).

### Predicting 3D models of CPP-pep11 interactions with HPV16 E6 and HPV18 E6 by AlphaFold2

The HPV16 E6 and HPV18 E6 proteins share nearly identical domain organization and a 57.04% sequence identity (Supplementary Figures S11A, S11B and S11C) [22, 53]. Structural studies showed that the HPV16 E6 protein contains a basic-hydrophobic pocket formed by its E6N and E6C zinc-binding domains together with a linker helix, which captures the helical LXXLL motif of E6AP. The E6/E6AP binding is stabilized through hydrophobic interactions between the E6 pocket and the leucine-rich LXXLL motif of E6AP, as well as electrostatic interactions involving acidic residues of E6AP and basic arginine residues of E6, especially the highly conserved R102 residue, which helps stabilize the entire complex (Supplementary Figure S11A) [14, 53], . Surface Plasmon Resonance (SPR) and NMR analysis showed that pep11 binds to the same E6 pocket as E6AP but with much stronger affinity (dissociation constant K_D_=2.3 ± 0.2 µM for E6APpep/E6 *versus* 34.0 ± 2.4 nM for pep11 soluble variant/E6) [22]. These findings support the idea that the E6 oncoprotein is a promising druggable target and suggest that its flexible binding cavity may accommodate peptides with diverse binding motifs.

To investigate how CPP-pep11 interacts with HPV16 and 18 E6 proteins, we employed AlphaFold2 via ColabFold platform [54, 55], which produces five structural models for each query and ranks them using confidence metrics including pLDDT (local structural accuracy), pTM (global structural accuracy), and ipTM (accuracy of intermolecular interfaces) [56]. High pLDDT (> 90), pTM (> 0.5), and ipTM (> 0.8) values indicate highly reliable structural predictions for the modelled structures [41, 55, 57–60].

### AlphaFold models of the HPV16 E6/CPP-pep11 complex

**Fig. 1:**
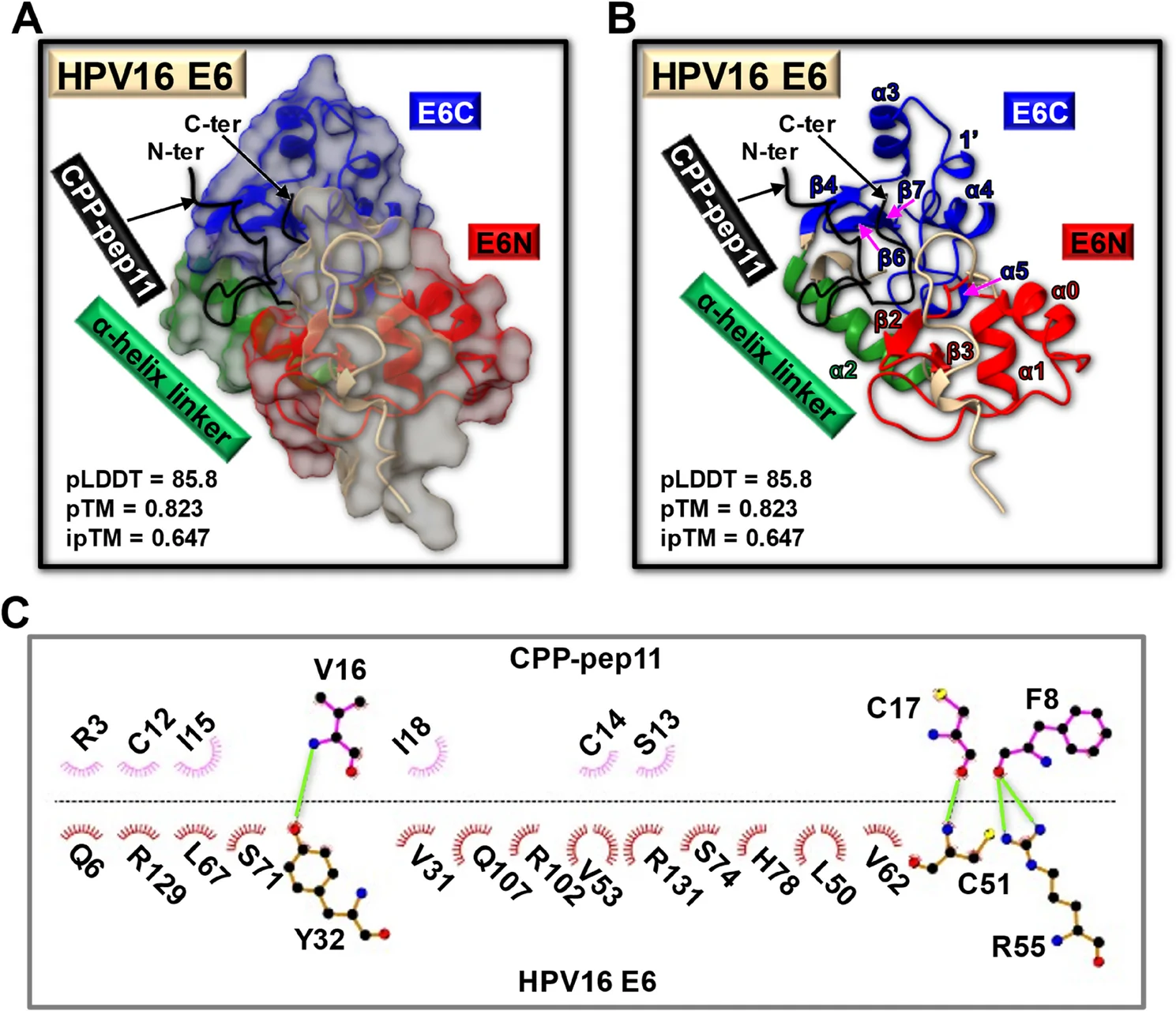
AF2 modelling of the HPV16 E6/CPP-pep11 complex. **A** Ribbon representation of the best AF2 [41, 42] model obtained for the HPV16 E6/CPP-pep11 complex. The E6N, α-helix linker and E6C regions of HPV16 E6 are coloured red, dark green and blue, respectively; the CPP-pep11 peptide (sequence: 1-RRRRRR-EFGSGCSCIVCIGLI-21) is shown in black. The surface of the HPV16 E6 is reported as well in the transparent drawing mode. The Figure has been generated with the software UCSF Chimera X (version 1.11.1) [61]. **B** Ribbon representation of the best AF2 [41, 42] model obtained for the HPV16 E6/CPP-pep11 complex. **C** 2D diagram of intermolecular interactions generated with LigPlot+ [46, 47] for the complex shown in (**A**). Carbon, Nitrogen, and Oxygen atoms are indicated by black, blue, and red spheres, respectively. H-bonds are represented by light green lines. To detect H-bonds the following maximum distance cut-offs were considered: 2.7 Å (H-acceptor) and 3.35 Å (donor-acceptor). Red and pink crescents with bristles are used for HPV16 E6 and CPP-pep11 residues (labelled with the one-letter amino acid code and sequence numbers) involved in further non-bonded interactions, respectively

The amino acid sequence of CPP-pep11 along with that of the HPV16 E6 protein were used as input query in ColabFold to generate the 5 best models of the CPP-pep11/HPV16 E6 complex. The best ranking prediction is presented in Fig. 1A. The peptide binds within the E6 deep pocket defined by the E6N and E6C domains forming a two walls-like structural organization with a floor provided by the α-helix linker (Fig. 1A and B). Peptide binding is stabilized by intermolecular contacts with several residues belonging mainly to E6N and linker helix regions. The interface between peptide and protein was analysed with the LigPlot+ software to generate 2D diagrams of intermolecular interactions (Fig. 1C) [46, 47]. Such analysis demonstrated that many HPV16 E6 residues, which have been shown to form the hydrophobic pocket and to interact with the LxxLL motif, are also part of the binding sites of CPP-pep11 (i.e., V31, Y32, L50, C51, V53, V62, L67, S74). Most residues contribute through non-bonded interactions with CPP-pep11, while Y32 and C51 form intermolecular H-bonds. S74 is critical for pep11–E6 binding, as its mutation selectively reduces interaction with soluble-enhanced pep11 [53]. Additional E6 residues forming the CPP-pep11 contact interface include H78, R129, R131 and R55. According to crystallography studies, these basic residues stabilize E6AP binding by forming a positively charged surface around the hydrophobic pocket that enables additional polar and electrostatic interactions with negatively charged E6AP residues [53]. In pep11, which contains only one negatively charged residue (E7), this basic patch mainly contributes to binding through intermolecular hydrogen bonds (i.e. R55) and non-bonded interactions (Fig. 1C).

R102 is part of the CPP-pep11 binding interface, and AlphaFold2 predicts a binding mode similar to LxxLL peptides on HPV16 E6, sharing the same site but with CPP-pep11 remaining largely disordered rather than helical (Fig. 1B). This model is consistent with cryo-EM structures of the E6/E6AP/p53 complex, and also shows S71 and Q107 (Supplementary Figures S12A, S12B, S12C, S12D), that in E6 are known to provide crucial intramolecular contacts between E6C and the α-helix linker region, to form new intermolecular contacts with CPP-pep11 residues I15 and I18 (Fig. 1B) [22].

Previous mammalian-two hybrid screening demonstrated that residues C6, C8, I9, C11 and I12 in pep11 (corresponding to C12, C14, I15, C17, and I18 in CPP-pep11) were key residues contributing to pep11 binding to E6 and that also the hydrophobic C-terminal peptide side was important for the interaction [23]. Indeed, residues C12, I15, C14, C17, I18 are part of the interaction surface in our best AlphaFold2 model of CPP-pep11/HPV16 E6 complex, together with R3, F8, S13 (Fig. 1C).

CPP-pep11 interacts with E6 primarily via its C-terminal region, where residues V16, C17, and F8 form intermolecular hydrogen bonds and additional residues engage in non-bonded contacts (Fig. 1). In contrast, the CPP sequence remains largely solvent-exposed and contributes minimally to binding, with only R3 participating at the interface, consistent with its role in maintaining peptide solubility while the hydrophobic C-terminus inserts into the E6 binding pocket (Fig. 1B).

The AlphaFold2 confidence metrics indicate a generally reliable model, with pLDDT values close to 90, slightly reduced due to the intrinsically disordered regions of the peptide and flexible loops in the protein. The E6 fold closely matches the experimental structure, confirming high accuracy for the protein core. In contrast, the ipTM score (0.6–0.8) suggests uncertainty in the precise peptide orientation within the binding site, consistent with the known flexibility of peptide–protein interactions that are often better represented as conformational ensembles. Similar analyses were conducted across the five best AlphaFold2 models (Supplementary Figures S13-S16).

The interaction features observed in the best AlphaFold2 model (Fig. 1) are consistent across the other four predictions (Supplementary Figures S13–S16). In all cases, CPP-pep11 adopts a disordered conformation and binds within a deep cavity formed by E6N, E6C, and the linker helix, with the CPP N-terminus remaining solvent-exposed. The binding site is predominantly hydrophobic, with additional stabilization from surrounding basic residues, especially arginines that provide secondary electrostatic contacts. Recurring hydrogen bonds include E6 R55 with peptide S13 and the highly conserved interaction between E6 C51 and peptide I15 across all models (Fig. 1C; Supplementary Figures S13B-S16B). In three models, a putative disulphide bond between E6 C51 and peptide C17 is also predicted, although this is not supported by experimental E6–peptide structures and remains speculative (Supplementary Figures S13, S15B, S16B). While pLDDT and pTM scores indicate overall good model quality, lower ipTM values highlight uncertainty in defining the precise peptide orientation, consistent with the inherent flexibility of short, disordered peptides (Fig. 1A and Supplementary Figures S13A-S16A). The root means square deviation (RMSD) comparisons confirm that structural variability is largely confined to the peptide, whereas the E6 scaffold remains highly consistent (Supplementary Figure S17).

Overall, AlphaFold2 predicts a model for the CPP-pep11/HPV16 E6 complex resembling experimental structures of HPV16 E6 in complex with E6AP [14, 53] and perfectly in line with previously published experimental studies on the interaction between optimized soluble pep11 peptide variant and HPV16 E16 [22].

### AlphaFold models of the HPV18 E6/CPP-pep11 complex

**Fig. 2:**
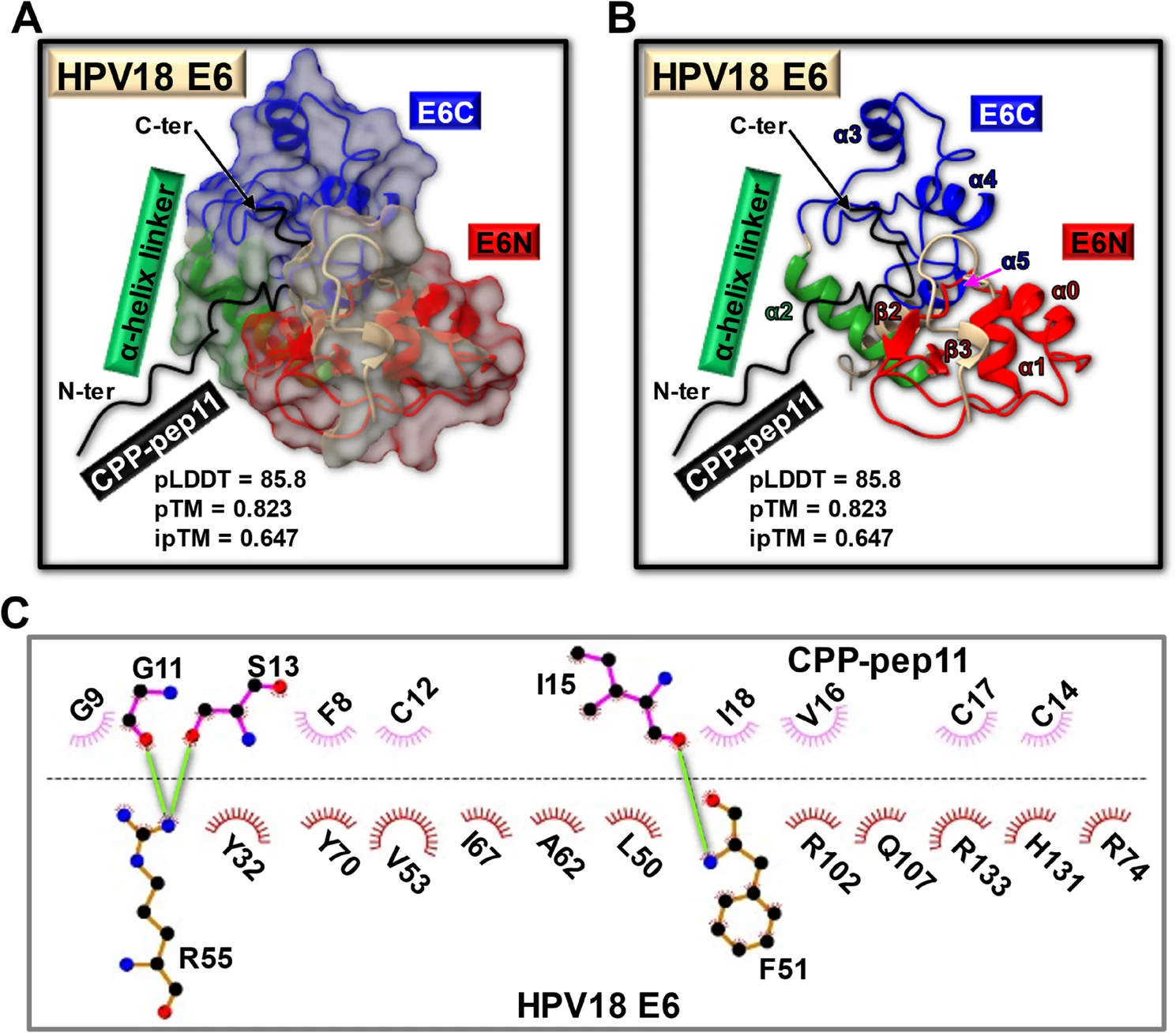
AF2 modelling of the HPV18 E6/CPP-pep11 complex. **A** Ribbon representation of the best AF2 [41, 42] model obtained for the HPV18 E6/CPP-pep11 complex. The E6N, α-helix linker and E6C regions of HPV18 E6 are coloured red, dark green and blue, respectively; the CPP-pep11 peptide (sequence: 1-RRRRRR-EFGSGCSCIVCIGLI-21) is reported in black. The surface of the HPV18 E6 is shown as well in the transparent drawing mode. The Figure has been generated with the software UCSF Chimera X (version 1.11.1) [61]. **B** Ribbon representation of the best AF2 [41, 42] model obtained for the HPV18 E6/CPP-pep11 complex. **C** 2D diagram of intermolecular interactions generated with LigPlot+ [46, 47] for the HPV18 E6/CPP-pep11 complex shown in (**A**). Carbon, Nitrogen, and Oxygen atoms are represented by black, blue, and red spheres, respectively. Intermolecular H-bonds are designated with solid light green lines. Maximum cut-offs for H-acceptor distances of 2.7 Å and donor-acceptor of 3.35 Å were set to find H-bonds. HPV18 E6 and CPP-pep11 residues involved in further non-bonded interactions are labelled with the one-letter amino acid code and residue number and represented by red and pink crescents with bristles, respectively

To investigate a potential interaction between HPV18 E6 and CPP-pep11, we used AlphaFold2 to model the complex and analysed intermolecular contacts with LigPlot+ [44, 46, 47, 60, 62]. As shown in Fig. 2, the best predicted structure closely resembles the HPV16 E6/CPP-pep11 complex and previously reported E6AP-bound structures, with CPP-pep11 adopting a largely disordered conformation within a conserved cleft formed by E6N, E6C, and the linker helix (Figs. 2; Supplementary Figures S18-S22). The CPP segment remains solvent-exposed, while the peptide core occupies a mainly hydrophobic pocket involving key residues (i.e., Y32, L50, F51, V53, R55, A62, I67, Y70, R74, R102, Q107, H131, R133), many of which overlap with the LxxLL-binding interface and are conserved or conservatively substituted between HPV16 and HPV18 (Fig. 2B). Among these residues, the Y32, L50, F51, V53, A62, I67, Y70 correspond to those that, according to crystallographic studies, are relevant for the interaction between HPV16 E6 and the LxxLL motif of the E6AP peptide [53]. The R102 represents the important residue for the stabilization of the E6N and E6C domains and peptide contacts, while surrounding basic residues (i.e., R55 and H131) contribute intermolecular H-bond and additional polar contacts (Fig. 2B). Notably, HPV18 E6 R74, corresponding to S74 in HPV16, may influence binding through steric effects, consistent with reported mutational data [22]. AlphaFold2 confidence score is high for the overall fold (pLDDT/pTM) but lower for peptide orientation (ipTM), reflecting intrinsic flexibility of the complex (Fig. 2A). Most interactions are hydrophobic, with a limited number of hydrogen bonds involving R102 and backbone carbonyl groups of G11/S13 and F51/I15 (Fig. 2C).

Alternative models show increased variability. The second best model shows that the interactions are mediated by side chains of HPV18 E6 arginine residues (i.e., R55, R74, R102, R133 corresponding in HPV16 to R55, S74, R102 and T133) (Supplementary Figures S19A, S19B). Interestingly, while the peptide CPP-pep11 N-terminal part largely moves outside the binding pocket, the inner region tends to assume a helical conformation encompassing residues S10-G19 (Supplementary Figure S19B). This type of structural binding topology closely matches the E6/E6AP complex [14]. The third model shows CPP-pep11 in a disordered binding mode, with the CPP N-terminus exposed and interactions dominated by non-bonded contacts (Supplementary Figures S20A, S20B). In the fourth model, R102 and F51 form hydrogen bonds with the peptide, which adopts a mostly flexible conformation with a short helical segment (I15–G19) and additional non-bonded interactions (Supplementary Figure S21A, B). The fifth model appears less reliable, showing an unrealistic peptide conformation with an atypical backbone geometry, suggesting a likely incorrect prediction (Supplementary Figure S22A, B). Overall, pairwise RMSD analysis highlights substantial structural variability across models, greater than that observed for the HPV16 E6 complex (Supplementary Figure S23A, B), pointing out a more flexible and potentially weaker HPV18 E6/CPP-pep11 interaction that will require experimental validation.

In conclusion, AlphaFold2 suggests that CPP-pep11 can bind both HPV16 and HPV18 E6, although the predicted peptide orientation is uncertain in both cases. The greater structural variability observed among the top HPV18 models compared to HPV16 suggests a weaker or less stable interaction, likely influenced by sequence differences such as the S74R substitution previously reported to affect the binding [22]. Nevertheless, the relatively good pLDDT values for the HPV18 complex indicate that a transient or weak interaction is still plausible, consistent with the peptide observed anticancer activity in HPV18-positive cell assays.

### Cellular uptake of CPP-pep11

**Fig. 3:**
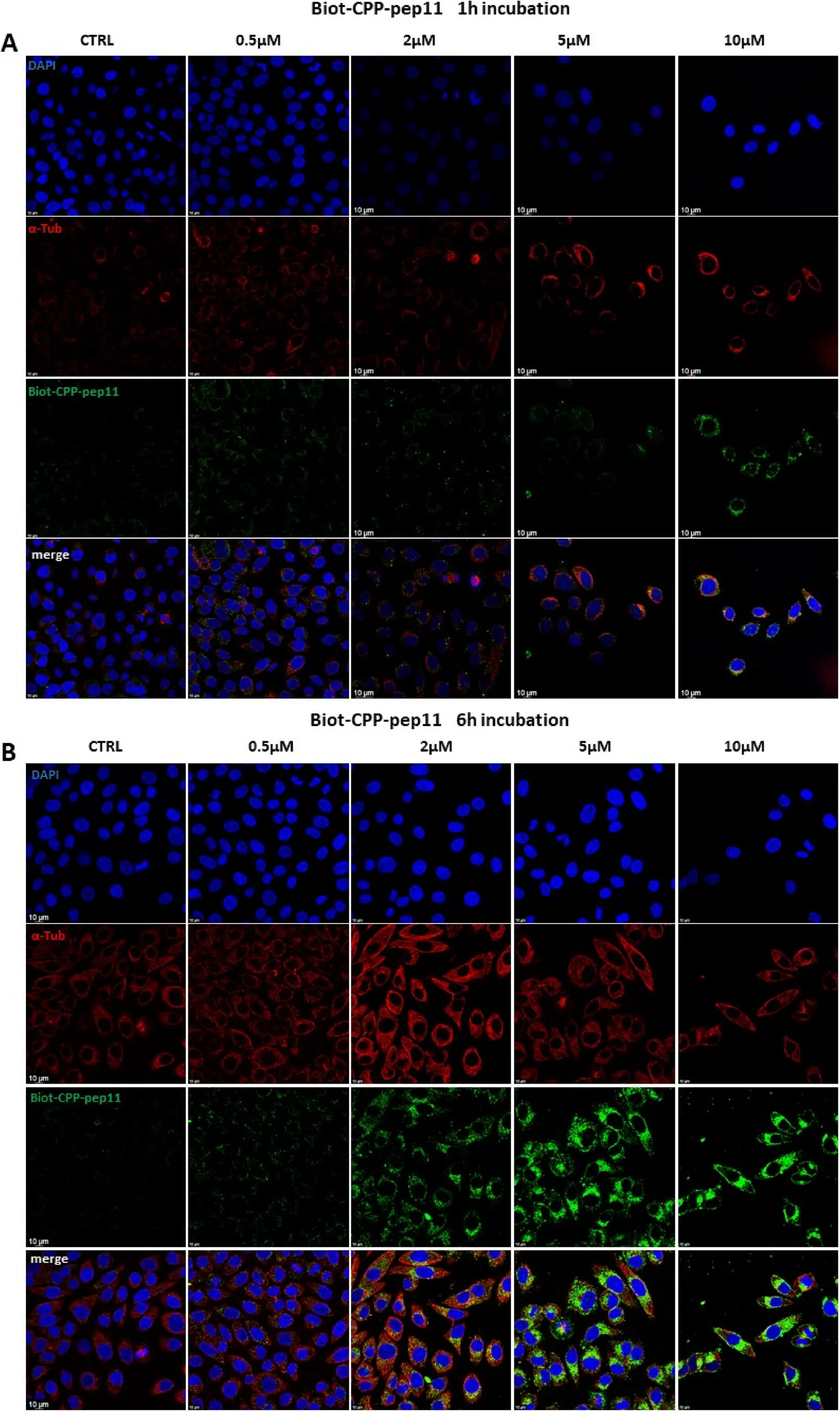
Internalization timing of CPP-pep11 in SiHa cell line. Representative immunofluorescence images of control SiHa cells and treated cells with biotinylated-CPP-pep11 (Biot-CPP-pep11) at different concentrations (0.5–2 − 5–10 µM) at 37 °C for 1 h (**A**) and 6 h (**B**). Cells were stained for Biot-CPP-pep11 (green); α-tubulin (red) and nuclei were counterstained with Hoechst 33342 (blue). Merged images are shown in the bottom panels. Incubation at 6 h induced a cytoplasmic accumulation of Biot-CPP-pep11. Scale bar: 10 μm

HPV16-positive SiHa and HPV18-positive C4-I cervical carcinoma cells were used to evaluate the internalization and subcellular distribution of CPP-conjugated peptides. Cells were seeded on glass-bottom 8-well µ-slides at 2 × 10⁴ cells/well and allowed to adhere for 24 h. Then, cells were incubated with Biot-CPP-pep11 or the scrambled control Biot-CPP-SCRpep11 at 0.5, 2, 5, and 10 µM for 1–6 h at 37 °C in 5% CO₂ in serum-free medium. Internalized peptides were detected using Streptavidin–Alexa Fluor 488. Cytoskeletal structures were stained with anti-α-tubulin antibody and nuclei were counterstained with Hoechst 33342 (Fig. 3). Peptides were observed to cross the cell membrane within 1 h of exposure in a dose-dependent manner (0.5–10µM) (Fig. 3A), with a stronger signal visible after 6 h, primarily in the cytoplasm (Fig. 3B). Immunofluorescence analysis showed that peptide uptake occurred even at 0.5 µM, with cytosolic accumulation increasing at higher concentrations, without causing membrane disruption. Biot-CPP-pep11 appeared as discrete cytosolic dots, suggesting uptake via endocytosis, and the absence of diffuse green fluorescence indicates limited endosomal escape. However, low levels of peptide in the cytosol cannot be excluded, as such signal may fall below the detection limit of confocal microscopy.

### Subcellular localization and stability of Biot-CPP-pep11

**Fig. 4:**
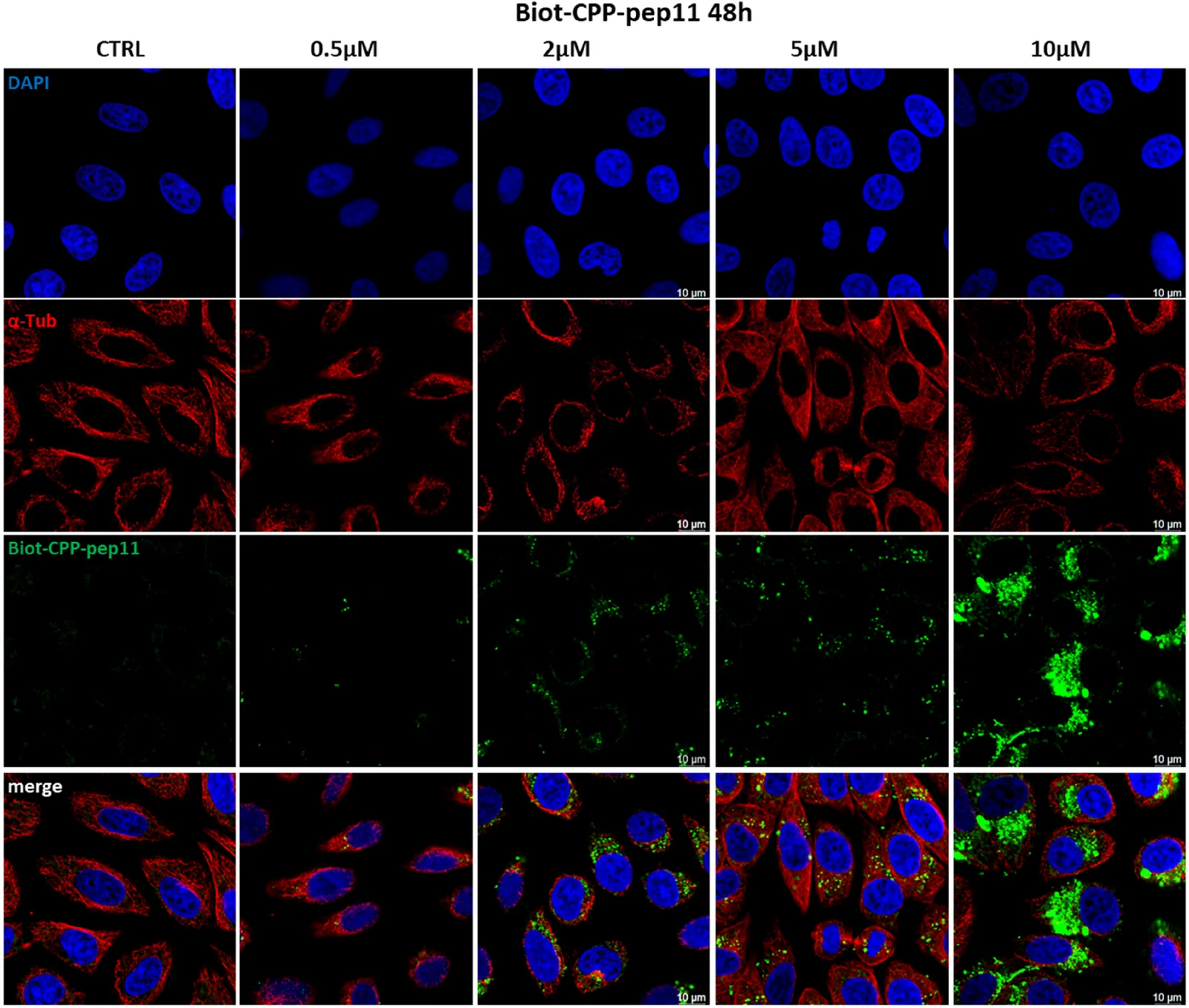
Cellular localization of Biot-CPP-pep11 in HPV16-positive SiHa cells. Representative immunofluorescence images of HPV16-positive SiHa cells untreated (control) or treated for 48 h with increasing concentrations (0.5, 2, 5, 10 µM) of Biot-CPP-pep11. Cells were stained for Biot-CPP-pep11 (green), α-tubulin (red), and nuclei (blue). Images acquired at 48 h show a dose-dependent accumulation of Biot-CPP-pep11 in the cytoplasm. Scale bar:10 μm

The intracellular distribution and stability of Biot-CPP-pep11 are critical for its efficient cellular delivery and biological effect [63]. To investigate these properties, SiHa and C4-I cells were treated with Biot-CPP-pep11 or Biot-CPP-SCRpep11 (0.5, 2, 5, 10 µM) for up to 48 h, followed by fixation and analysis by confocal microscopy. Biot-CPP-pep11 predominantly localized in the cytoplasm at 10 µM concentration (Fig. 4 and Supplementary Figure S24). At the higher concentration (20 µM), the peptide accumulated prevalently in the nucleus in SiHa cells (Supplementary Figure S25), suggesting dose-dependent transport through endocytic vesicles and subsequent nuclear release. In contrast, Biot-CPP-SCRpep11 entered cells at lower concentrations but remained largely outside the plasma membrane at higher concentration (5 and 10 µM) (Supplementary Figure S26A).

A further control was carried out under the same experimental conditions to evaluate the subcellular localization of the peptide Biot-CPP-OVA₂₅₇₋₂₆₄, which is more hydrophilic and less prone to aggregate compared to Biot-CPP-SCRpep11. However, after 48 h of incubation, no detectable fluorescence signal was observed (Supplementary Figure S26B), likely due to the limited interaction of the peptide with the cell membrane, resulting in its removal during the washing steps. To further support this hypothesis, cells were incubated with Biot-CPP-OVA₂₅₇₋₂₆₄ and Streptavidin-Alexa Fluor 488 for 1 h and subsequently imaged by confocal fluorescence microscopy prior to fixation. Under these conditions, Biot-CPP-OVA₂₅₇₋₂₆₄ displayed a strong fluorescence signal mostly localized at the cell membrane. Notably, this signal was substantially reduced following fixation, washing, nuclear staining, and mounting procedures, further suggesting that the peptide was mainly associated with the plasma membrane rather than being efficiently internalized (Supplementary Figure S27).

SiHa cells treated with Biot-CPP-pep11 showed positive signals after 96 h of culture, indicating that the peptide remains stable within the intracellular environment with minimal degradation. (Supplementary Figure S28).

**Fig. 5:**
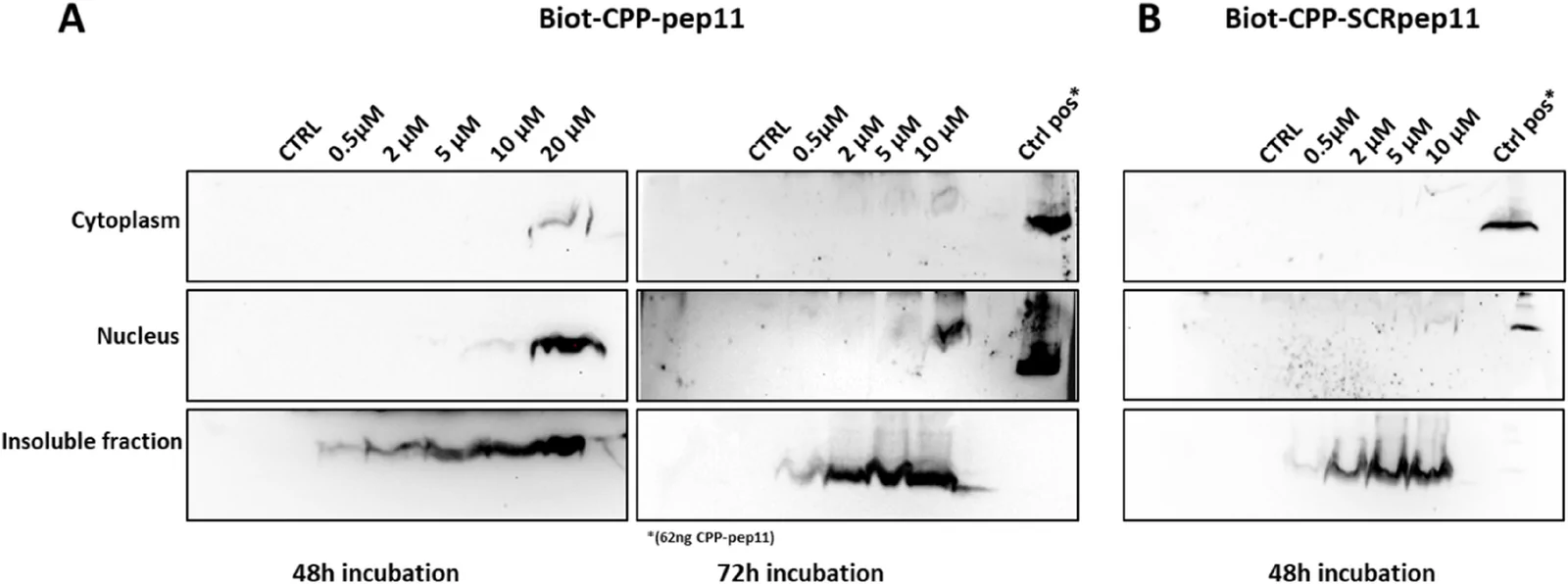
Subcellular distribution of CPP-pep11. **A** Cytoplasmic and nuclear extracts from untreated (control) SiHa cells or cells treated for 48 and 72 h with Biot-CPP-pep11 (0.5-2-5-10-20 µM) or (**B**) Biot-CPP-SCRpep11 (0.5–2 – 5–10 µM) were subjected to Western blot analysis. Membranes were probed with anti-biotin antibody to detect Biot-CPP-pep11 and Biot-CPP-SCRpep11. Representative blots from three independent experiments are shown. Chemiluminescent signals were detected using the iBright™ CL1500 Imaging System (Thermo Fisher Scientific)

Peptide intracellular distribution was assessed by western blotting on cytoplasmic or nuclear cell extracts obtained after 48-hour of treatment with increasing doses of peptide (0.5µM, 2µM, 5µM, 10 µM and 20µM), (Fig. 5). The analysis confirmed nuclear localization of Biot-CPP-pep11, with a nuclear signal detectable at 10 µM and stronger at 20 µM and still visible after 72 h (Fig. 5A). In contrast, Biot-CPP-SCRpep11 was absent from both nuclear and cytoplasmic fractions, with most of the peptide detected in the insoluble fraction, likely due to aggregation resulting from by the amphipathic, positively charged amino acid organization of the peptide sequence (Fig. 5B). On the other end, the different order of amino acids in CPP-pep11 likely contributed to its improved solubility and reduced aggregation compared with the scrambled control.

### Biot-CPP-pep11 cellular uptake mechanism

**Fig. 6:**
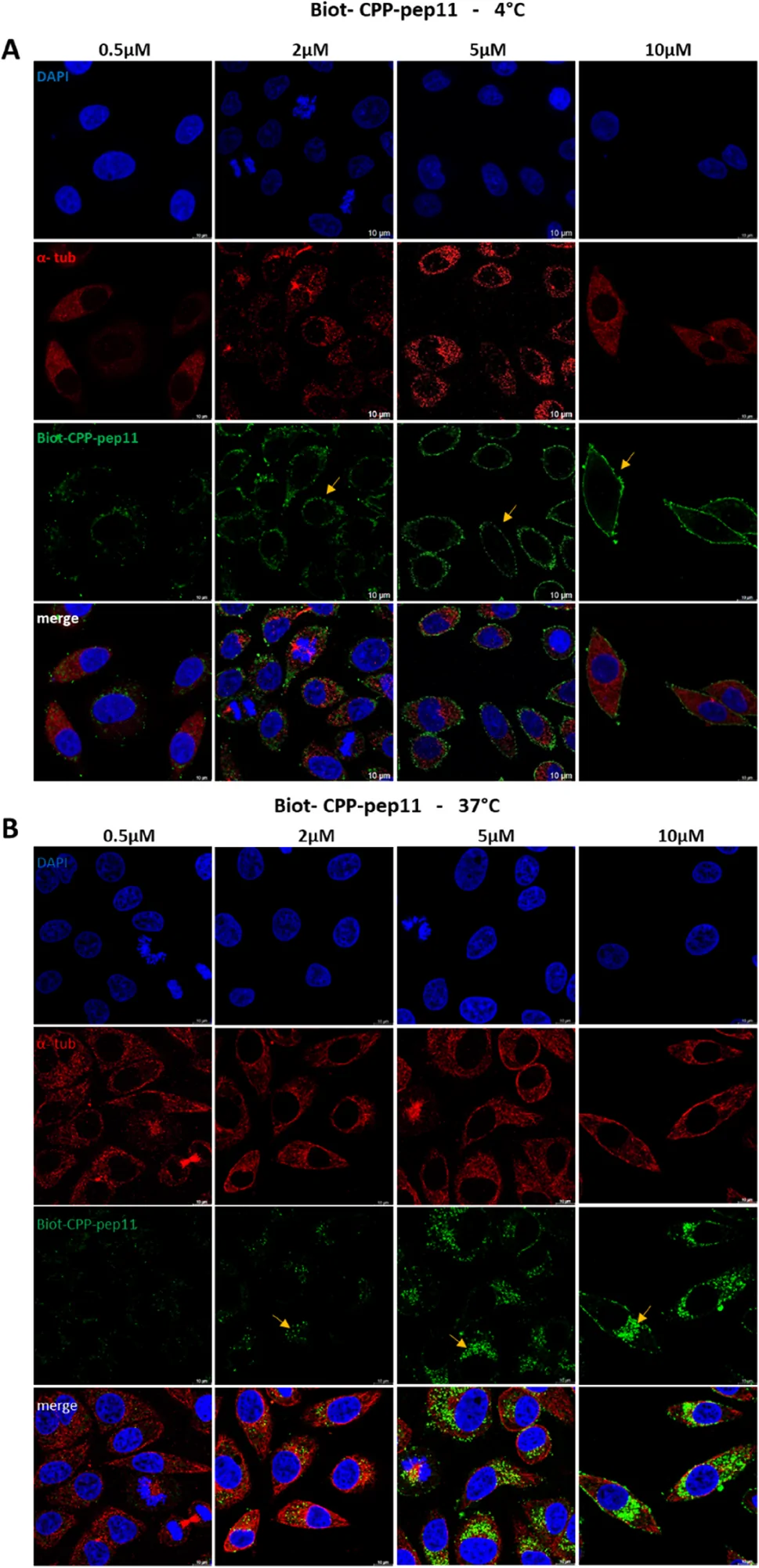
Identification of CPP peptide entry mechanism. Images of SiHa cells incubated with Biot-CPP-pep11 for 6 h at 4 °C (**A**) or 37 °C (**B**). Cells were then fixed and stained for the peptide (green), α-tubulin (red), and nuclei (blue). Incubation at 4 °C markedly reduced intracellular peptide signal compared to 37 °C (yellow arrows), indicating an energy and temperature-dependent uptake. Scale bar: 10 μm

Cationic CPPs are known to interact with anionic components of the plasma membrane, such as phosphate groups, and can translocate into cells via direct pore formation or other mechanisms [29, 64]. To elucidate the uptake mechanism of Biot-CPP-pep11, cells were seeded for 24 h, then treated with increasing peptide concentrations (0.5–10µM) and incubated at either 4–37 °C for 6 h. Confocal microscopy analysis demonstrated that Biot-CPP-pep11 uptake was strongly reduced at 4 °C, with the peptide signal mainly restricted to the cell surface, closely outlining the plasma membrane (Fig. 6A, green fluorescence). In contrast, Biot-CPP-pep11 efficiently penetrated the plasma membrane at concentrations as low as 0.5 µM under 37 °C culture conditions (Fig. 6B). These results indicate that Biot-CPP-pep11 uptake is primarily energy-dependent.

### Anti-proliferative effect of CPP-pep11 on cervical cancer cells

CPP-pep11 is expected to block the E6/E6AP interaction by competing for the LxxLL motif, thereby stabilizing p53 and inducing apoptosis to reduce proliferation and viability of HPV-positive cancer cells. To assess the dose-dependent effect on proliferation, SiHa and C4-I cells were treated with increasing peptide concentrations and monitored using a real-time cell index assay, which measures cell adhesion to the substrate via impedance sensing. Culture conditions in micro wells were first optimized for both cell lines (Supplementary Figure S29A, B). Cells were plated at 2.5 × 10³ per well and treated as described in Materials and Methods. Proliferation was monitored for 72 h following peptide treatment at 0 µM (negative control), 0.5 µM, 2 µM, 5 µM, 10 µM, and 20 µM.

**Fig. 7:**
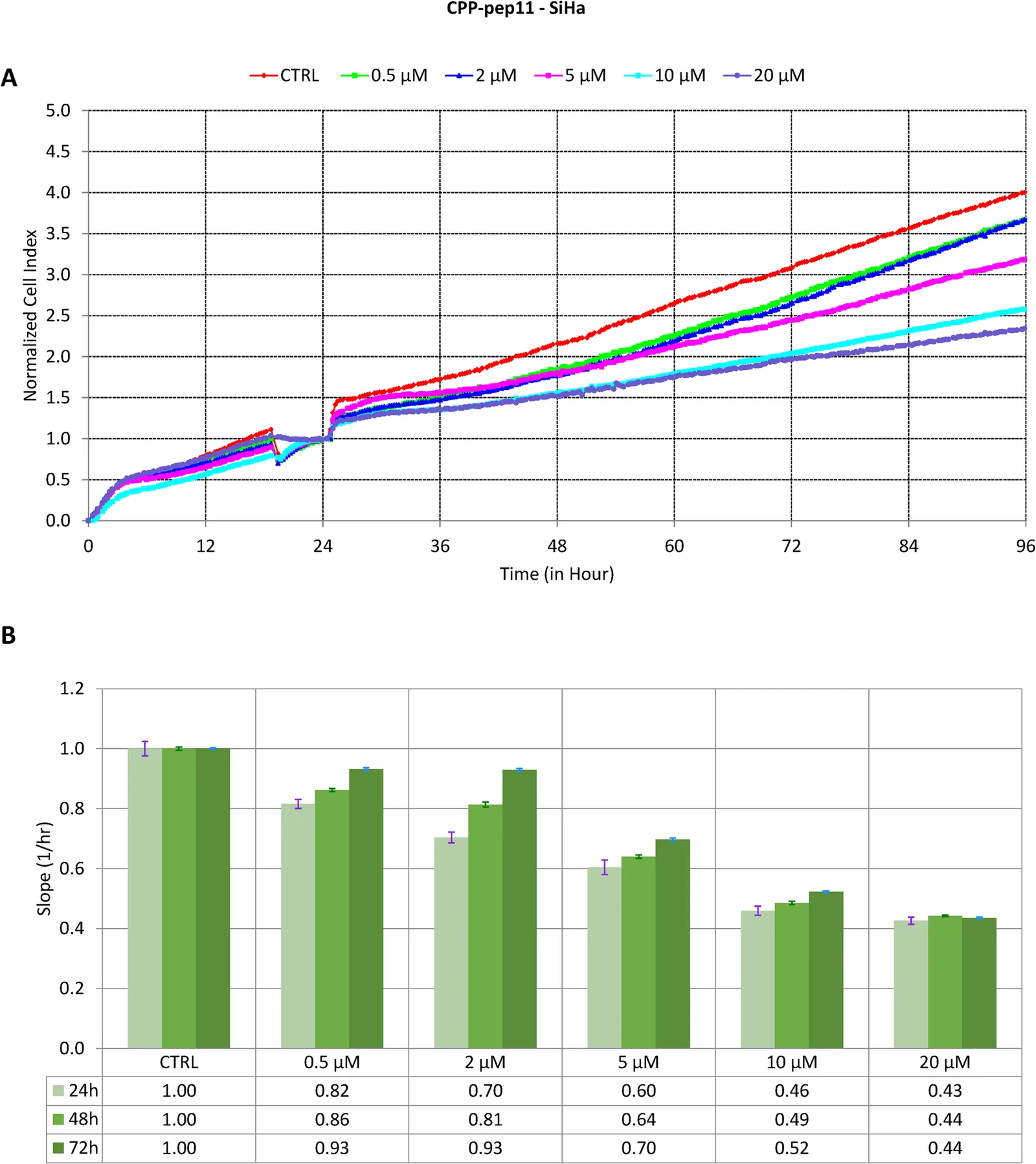
Real-time monitoring of cell proliferation in SiHa cell line by cell index assay. Cells were seeded in E-plates and allowed to adhere for 24 h before treatment with CPP-pep11 at the indicated concentrations (0, 0.5, 2, 5, 10, and 20 µM). Cell proliferation was monitored using the xCELLigence RTCA System, and impedance values (expressed as cell index) were recorded every 15 min for 96 h. **A** Cell index values were normalized to the time point immediately after serum addition. **B** Relative growth was calculated and expressed as fold change compared to untreated controls. Data are presented as mean ± SD of two independent experiments performed in quadruplicate

**Fig. 8:**
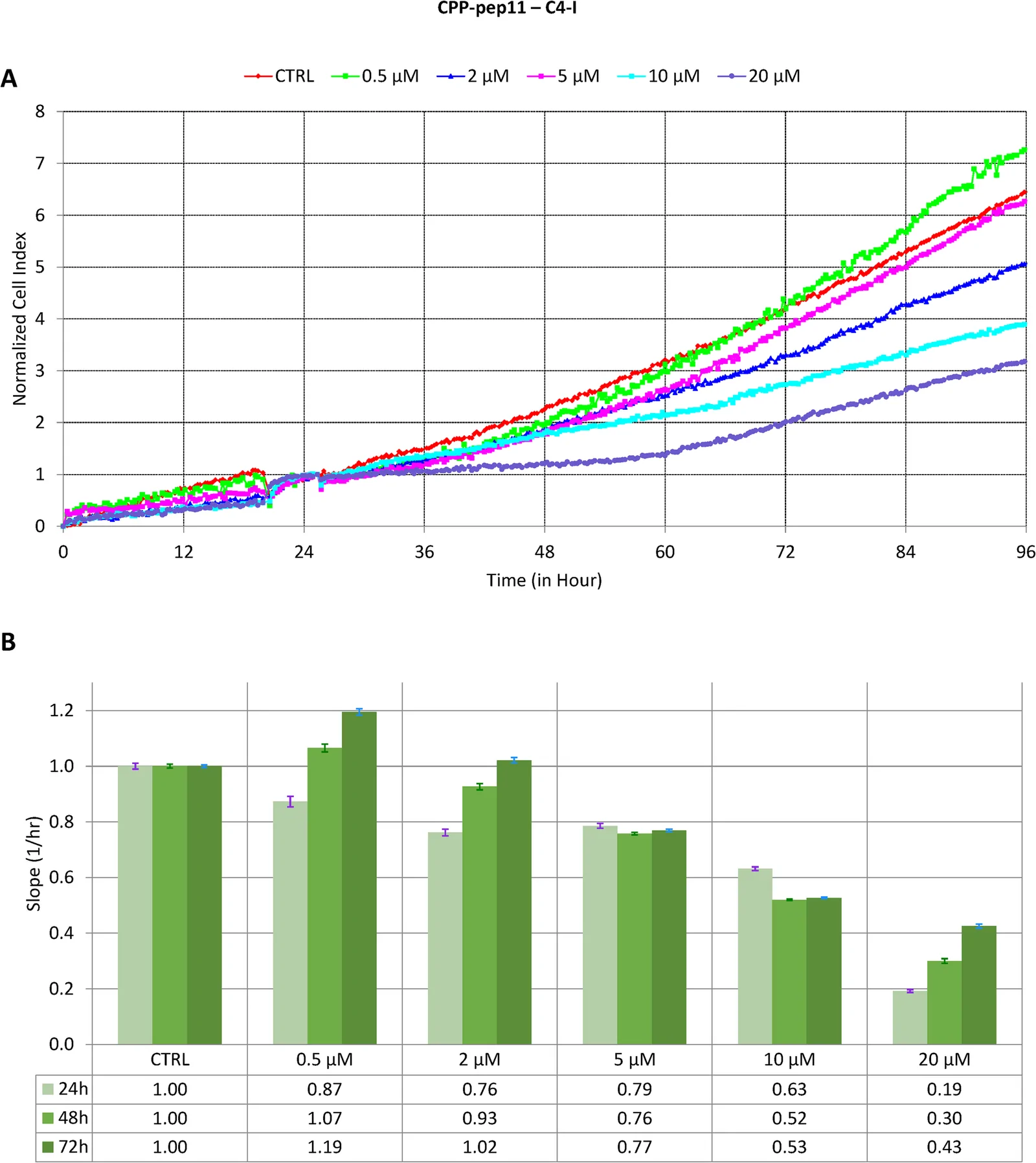
Real-time monitoring of cell proliferation in C4-I cell line by cell index assay. Cells were seeded in E-plates and allowed to adhere for 24 h before treatment with CPP-pep11 at the indicated concentrations (0, 0.5, 2, 5, 10, and 20 µM). Cell proliferation was monitored using the xCELLigence RTCA System, and impedance values (expressed as cell index) were recorded every 15 min for 96 h. **A** Cell index values were normalized to the time point immediately after serum addition. **B** Relative growth was calculated and expressed as fold change compared to untreated controls. Data are presented as mean ± SD of two independent experiments performed in quadruplicate

Proliferation curves and their slopes, following CPP-pep11 and CPP-SCRpep11 treatment, and derived from cell index measurements, are shown in Figs. 7 and 8 and in Supplementary Figures S30 and S31, respectively. CPP-pep11 produced a dose-dependent reduction in proliferation, with approximately 50% inhibition at 10 and 20 µM, reflected by a plateau in growth between 48 and 72 h (Fig. 7A). Importantly, CPP-pep11 did not induce significant toxicity in SiHa cells, even at the highest concentrations (Fig. 7B). In contrast, CPP-SCRpep11 caused a rapid decline in proliferation at higher concentrations, with an 80–90% reduction in growth rate at 10 and 20 µM within 24 h, by exhibiting a cytotoxic effect (Supplementary Figure S30A, B). Similarly, CPP-pep11 exerted a marked dose-dependent anti-proliferative effect in C4-I cells. At the highest concentration (20 µM), CPP-pep11 reduced cell proliferation by approximately 70–80% within the first 24 h. At intermediate concentrations (5 and 10 µM), cell proliferation was significantly reduced compared with untreated controls, whereas no appreciable effect was observed at the lowest concentration (Fig. 8A). Analysis of the proliferation rate further showed a dose-dependent decrease, with 5 and 10 µM CPP-pep11 producing an almost complete flattening of the growth curve (Fig. 8B). Although cells treated with 20 µM CPP-pep11 exhibited a partial recovery of proliferative activity after the initial inhibition, the overall proliferation rate remained markedly lower than that of control cells throughout the experiment.

As observed in SiHa cells, treatment with the scrambled control peptide (CPP-SCRpep11) also reduced C4-I cell proliferation at higher concentrations, with approximately a 50% decrease in proliferation at 5 µM and almost complete inhibition of growth at 20 µM (Supplementary Figure S31A, B).

Since CPP-SCRpep11 was even more toxic at 20 µM, a maximum concentration of 10 µM was used in subsequent experiments.

CPP-pep11 reduced cell viability in SiHa and C4-I cells in a dose-dependent manner, with a pronounced effect at 72 h at the highest concentration for both lines (Supplementary Figures S32). Cell viability started to decline at 5 µM and decreased markedly at the highest concentration. Treatment with the scrambled peptide produced a similar effect in both SiHa and C4-I cells, with a more pronounced reduction in cell viability observed at 5 and 10 µM, consistent with the findings from the real-time cell index assay.

**Fig. 9:**
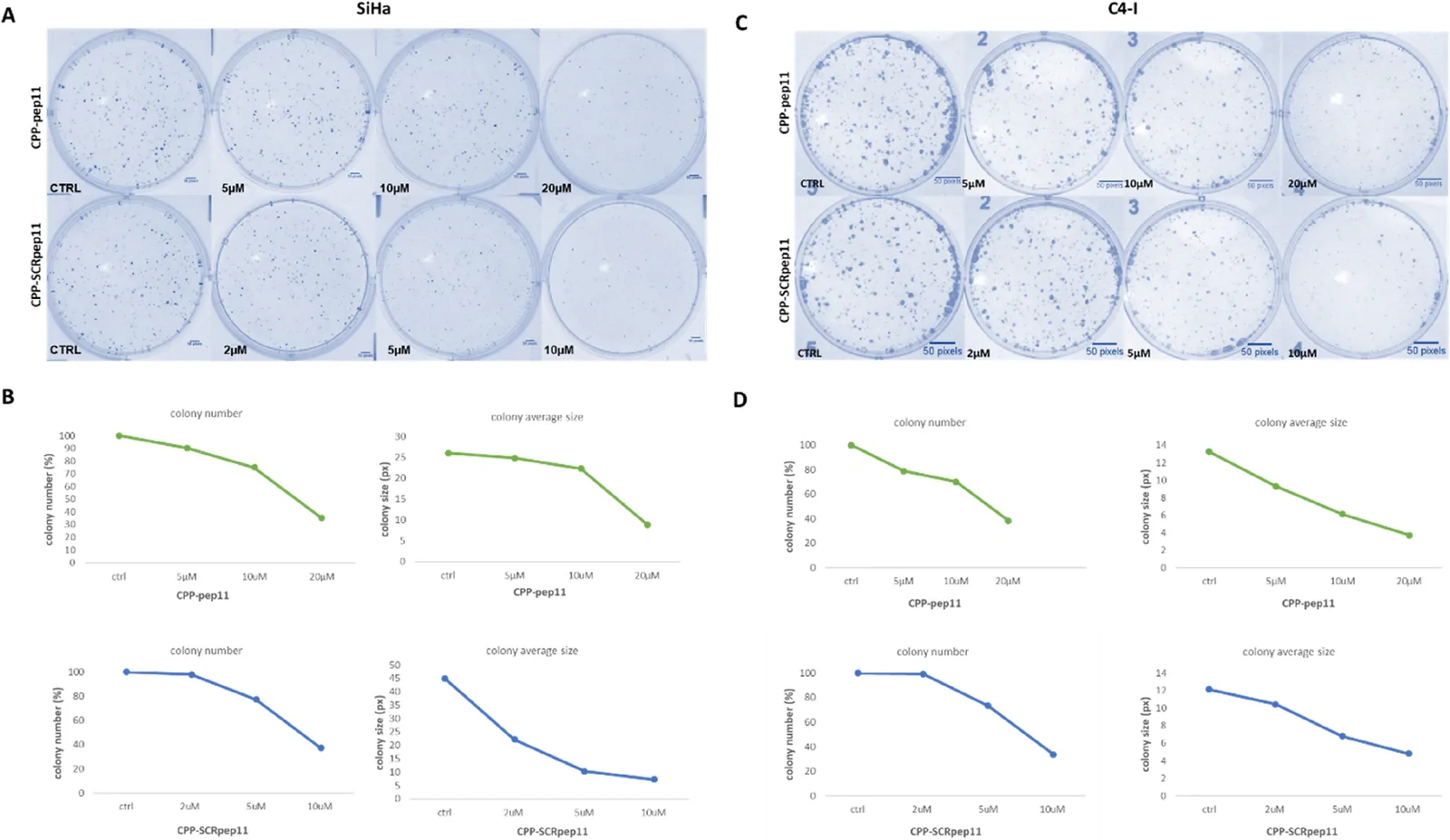
2D clonogenic assay in HPV16 (SiHa) and HPV18 (C4-I) cells upon CPP peptides treatment. Cells were seeded at low density and treated with the indicated concentrations of CPP-pep11 and CPP-SCRpep11 (**A** and **C**). After 10–14 days, colonies were fixed and stained with crystal violet. The green curves in figures **B** and **D** represent the percentage of colonies normalized to the control condition (100%) and the mean colony size for each sample at different concentrations of CPP-pep11. The blue curves in the bottom of panel **B** and **D** show the percentage of colonies normalized to the control condition (100%) and the mean colony size for each sample at different concentrations of CPP-SCRpep11. The number and the area of colonies was reduced at increasing concentrations of CPP peptides

Next, the colony-forming ability of SiHa and C4-I was assessed by clonogenic assay. Cells were seeded in six-well plates and allowed to grow. On day five, they were treated with CPP-pep11 (5–20 µM) or CPP-SCRpep11 (2–10 µM), followed by a second treatment on day eight, followed by an additional four days of culture. Colonies were stained with crystal violet and counted (Fig. 9A, C). The treatment with CPP-pep11 resulted in a clear dose-dependent reduction in colony number and colony size as quantified by ImageJ analysis (Fig. 9B, D, green curves). Specifically, the addition of 5 µM, 10 µM, and 20 µM CPP-pep11 caused a reduction of mean colony counts to 664 (90% of control), 551 (75%), and 258 (35%), respectively, compared to control samples (mean colony count 735). These findings demonstrate a strong dose-dependent inhibitory effect of CPP-pep11 on colony number and morphology. In the C4-I cell line, clonogenic capacity followed a similar trend to that observed in SiHa cells, however, C4-I cells appeared more sensitive to CPP-pep11 treatment, exhibiting a reduction in colony area already at 5 µM. In contrast, treatment with CPP-SCRpep11 in SiHa and C4-I cells markedly reduced colony size, indicating that significant cytotoxic effects occurred at the highest doses (Fig. 9B, D, blue curves).

**Fig. 10:**
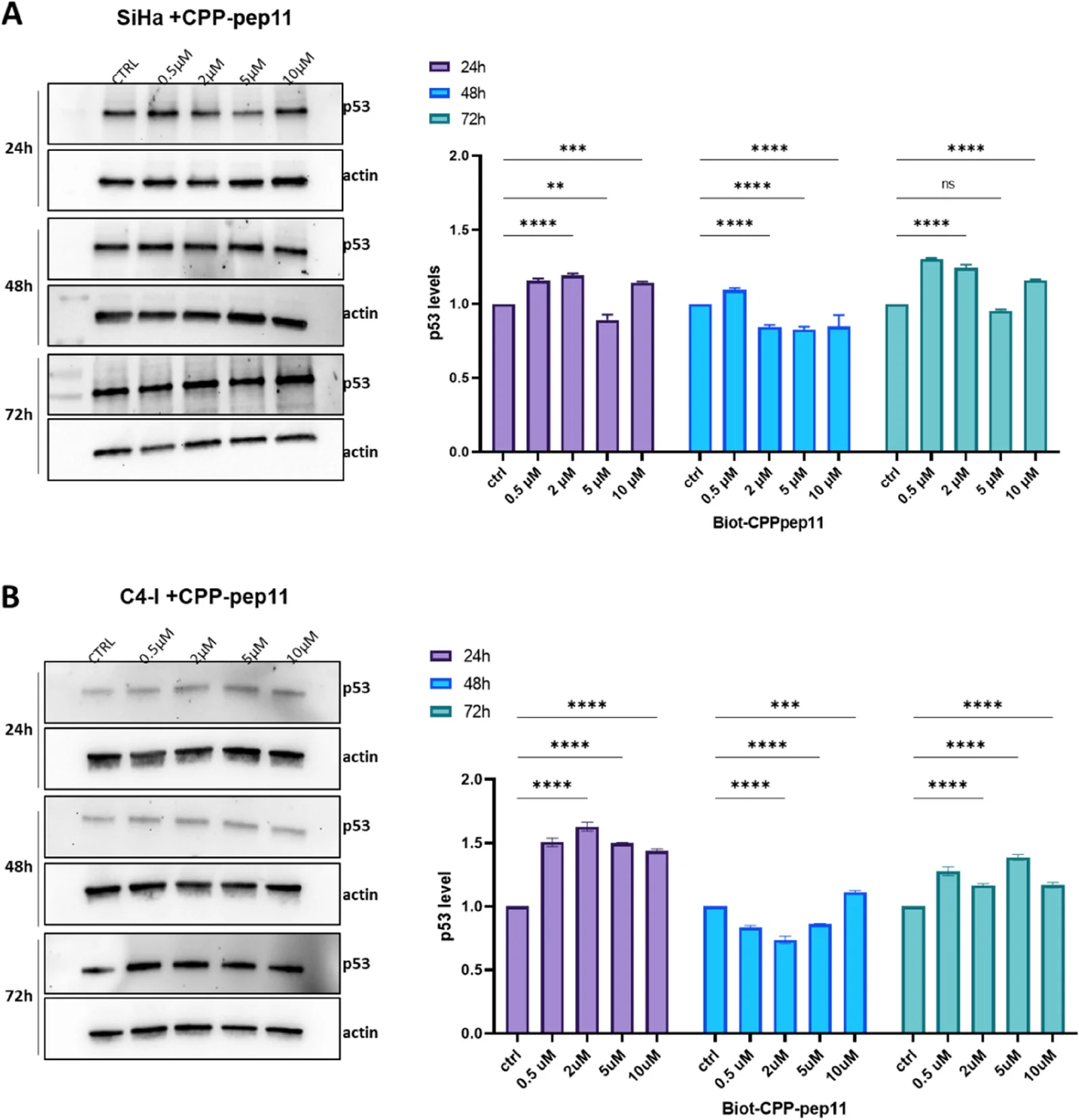
CPP-pep11 promotes p53 stabilization in SiHa and C4-I cells. Western blot analyses showing p53 protein levels in SiHa (**A**) and C4-I (**B**) cell lines following CPP-pep11 treatment at 24, 48, and 72 h. Densitometric quantification is presented as bar graphs, showing a time-dependent increase in p53 expression. Values were normalized to untreated control cells

Biot-CPP-pep11 is designed to bind the HPV E6 by competing with E6AP, thereby preventing p53 ubiquitination and degradation. Consistent with this mechanism, treatment of SiHa and C4-I cells with increasing concentrations of CPP-pep11 led to a dose-dependent accumulation of p53. In Fig. 10 are presented representative western blot analyses showing p53 expression in SiHa and C4-I cells treated with increasing concentrations of CPP-pep11 for 24, 48, and 72 h. In SiHa cells, CPP-pep11 treatment resulted in a time- and dose-dependent increase in p53 expression, particularly evident at intermediate concentrations (0.5-2 µM) and at 48 h and 72 h post-treatment. At higher concentrations (5–10 µM), p53 levels showed variable regulation, possibly reflecting additional cellular stress responses. Similarly, in C4-I cells, CPP-pep11 treatment induced an overall increase in p53 levels in a dose- and time-dependent manner, consistent with functional interference with E6 activity. Densitometric quantification, normalized to the respective loading controls and expressed relative to untreated controls (set to 1), confirmed the restoration of p53 following CPP-pep11 treatment. Collectively, these results support the hypothesis that CPP-pep11 disrupts E6-mediated p53 degradation, resulting in stabilization and accumulation of p53 in HPV-positive cervical cancer cells.

**Fig. 11:**
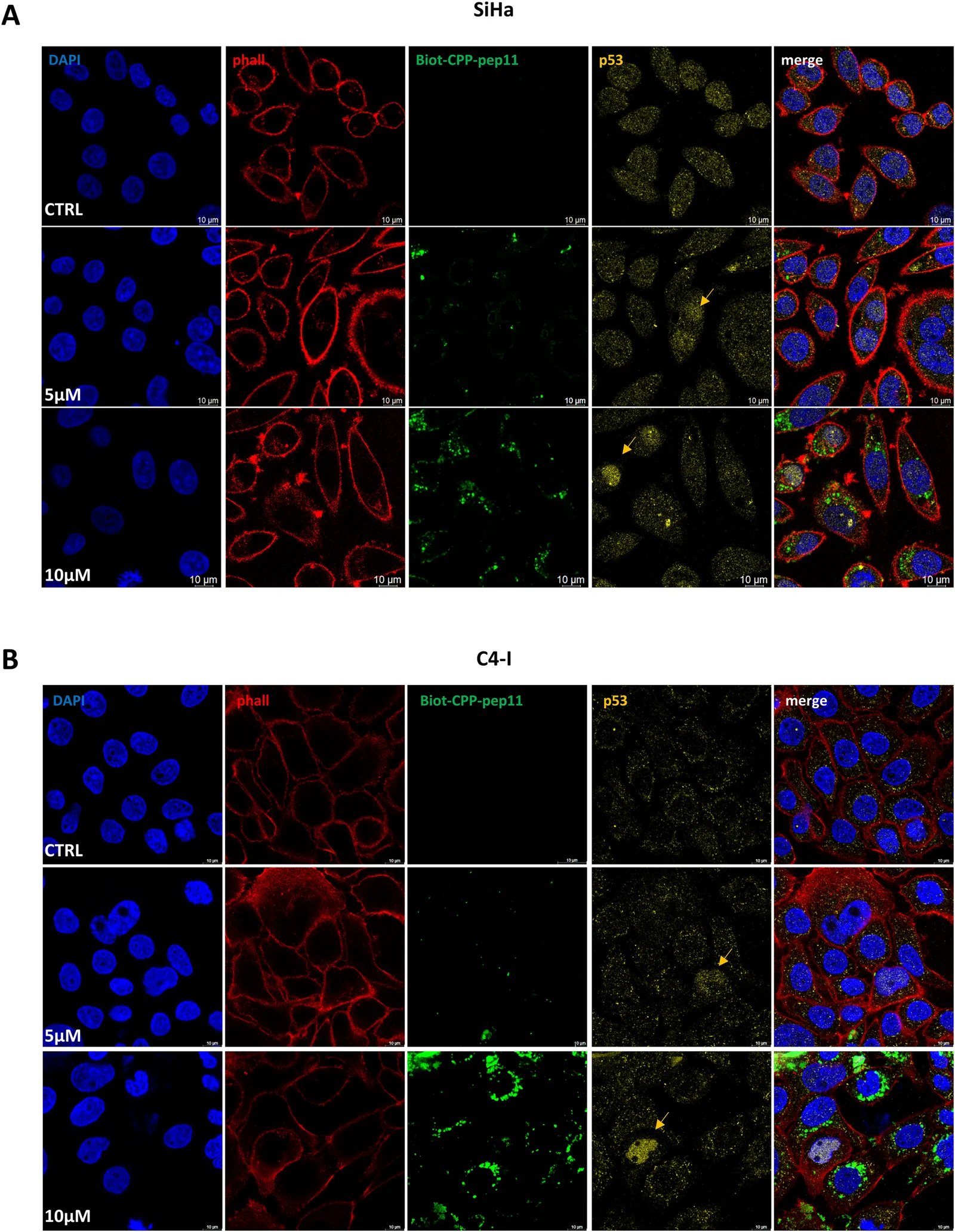
CPP-pep11 promotes p53 nuclearization in SiHa (**A**) and C4-I cell lines (**B**). Representative immunofluorescence images of SiHa cells untreated (control) or treated for 48 h with increasing concentrations of Biot-CPP-pep11 (5 and 10 µM). Cells were stained for Biot-CPP-pep11 (green), phalloidin (red), p53 (yellow) and nuclei (blue). Images acquired at 48 h show a dose-dependent accumulation of p53 in nuclear compartment. Scale bar: 10 μm

In Fig. 11 representative immunofluorescence images of HPV16-positive SiHa (Fig. 11A) and HPV18-positive C4-I (Fig. 11B) stained with Hoechst 33342 (blue), phalloidin (red), Strep-488 (green; detecting Biot-CPP-pep11), and p53 (yellow), along with merged images are shown. Biot-CPP-pep11 fluorescence is clearly detectable in treated samples, predominantly in the cytoplasmic and perinuclear regions, demonstrating efficient intracellular uptake of Biot-CPP-pep11 in both SiHa and C4-I cells. Importantly, p53 immunostaining revealed enhanced nuclear accumulation in treated cells compared to untreated controls. Merged images confirm the coexistence of intracellular peptide signal and elevated nuclear p53 staining within the same cells. Overall, these findings demonstrate effective cellular internalization of Biot-CPP-pep11 in both SiHa and C4-I cells and show associated stabilization and nuclear accumulation of p53, consistent with inhibition of E6-mediated p53 degradation.

## Discussion

In this study, we designed and characterized cell-penetrating peptide, namely CPP-pep11, derived from a previously described HPV16 E6-binding moiety to improve its solubility, cellular uptake, and functional interference with E6-mediated p53 degradation. Covalent attachment of a polyarginine CPP to the N-terminal end of pep11 markedly improved peptide solubility, enabling the preparation of stable aqueous solutions. The Boc synthesis strategy, rather than the more commonly used Fmoc approach, was required to achieve high peptide yields and minimize aggregation during synthesis [65, 66]. In the original pep11, the presence of three cysteine residues, which are essential for E6 binding, may cause aggregation due to the formation of disulphide bonds and altered protein structure [23, 67]. We verified that N-terminal conjugation of the CPP sequence to pep11 results in a peptide that does not undergo intra- or intermolecular disulphide bond formation, even after 72 h of air oxidation using DMSO solution or PBS solution [50]. These findings indicate that the limited aggregation observed during prolonged incubation arises from non-covalent intermolecular interactions rather than disulphide bond formation, preserving the structural integrity required for E6 recognition [68]. HPLC analysis was used to investigate both cysteine oxidation and protein aggregation in aqueous solution. Mass spectrometry analysis confirmed that the aggregation is not derived from cysteines oxidation. Moreover, NMR analyses of the peptides demonstrate that, under experimental conditions that favour non aggregated peptide forms, CPP-pep11 and CPP-SCRpep11 are characterized by disordered conformations that are not influenced by the presence of the biotin tail (Supplementary Figures S9 and S10). A scrambled peptide (CPP-SCRpep11) obtained via random shuffling of the original sequence pep11 has been used as negative control. Because CPP-pep11 was based on a relatively insoluble pre-existing peptide sequence, it may serve as a useful model for developing inhibitory molecules targeting the HPV E6 interactions [22, 69–72].

Previous studies have assessed the specificity of the interaction towards either HPV16 E6 and/or HPV18 E6. Binding studies were performed in both yeast and mammalian cells and revealed that the E6APpep (i.e., an 18 mer peptide sequence encompassing the binding site of E6AP for HPV E6) could interact in the yeast model with both HPV16 and HPV18 [22, 23]. Previous studies demonstrated that pep11 binds efficiently to HPV16 E6 and less efficiently to HPV18 E6 in yeast. However, in mammalian cells (human cervical cancer cells), an interaction was detected only with HPV16 E6, indicating that pep11 displays high selectivity for HPV16. In the present study, we evaluated the potential interaction of CPP-pep11 with HPV16 E6 and HPV18 E6 using AlphaFold2 modelling. Although the predicted binding affinity for HPV18 E6 was lower than that for HPV16 E6, the comparable biological activity observed experimentally suggests that peptide efficacy in living cells is influenced by factors beyond predicted structural affinity alone.

The CPP-pep11 exhibited enhanced hydrophilicity in aqueous solution and efficient cellular uptake without organic solvents. It entered HPV-positive cervical cancer cells in a dose- and time-dependent manner, showing cytoplasmic localization at concentrations as low as 0.5 µM, with accumulation increasing at higher doses and partial nuclear localization at 10–20 µM. Cellular internalization may occur through energy-independent processes or energy-dependent pathways, including endocytosis. Notably, incubation at low temperatures (4 °C) is widely recognized to inhibit energy-dependent uptake processes, such as endocytosis [73–75]. CPP-pep11 uptake was strongly reduced at 4 °C, indicating energy-dependent internalization, likely via endocytosis. The endocytic pathway is the primary mechanism for CPP uptake, leading to peptide sequestration within endosomes and lysosomes [76]. Consistent with these findings, several studies have reported the effectiveness of positively charged, arginine-rich CPPs in facilitating intracellular cargo delivery, largely due to their strong capacity to escape from endosomal compartments [72, 77, 78].

In addition, intracellular processes such as proteolysis can influence the overall accumulation of CPPs within cells [79–81]. In our study, immunofluorescence and western blot analyses performed on cells treated with CPP-pep11 for 6 h and maintained in culture for extended periods (up to 96 h) demonstrated that the peptide remained stable and progressively accumulated in the nucleus. Conversely, other studies have shown that peptides often require modifications at N- and C-termini, incorporation of non-natural amino acids, and peptide cyclization to enhance their stability [81–84]. For example, dimeric fluorescent Tat achieves stability up to 48 h through the incorporation of D-amino acids. Sustained intracellular retention of CPP-pep11 is particularly advantageous for therapeutic applications because prolonged peptide availability reduces the need for repeated administration while allowing continuous inhibition of E6 function.

Functionally, CPP-pep11 exhibited potent, dose-dependent anti-proliferative effects in HPV16-positive SiHa and HPV18-positive C4-I cells. Real-time cell index assays demonstrated a significant inhibition of cell proliferation at concentrations between 5 and 10 µM, without evidence of overt cytotoxicity. Although CPP-pep11 reduced proliferation in both cell lines, the inhibitory effect was more pronounced in C4-I than in SiHa cells. This differential response suggests that the greater sensitivity of C4-I cells may reflect intrinsic molecular differences between the two models, particularly with respect to HPV E6 expression and their dependence on E6-driven oncogenic signalling.

Indeed, SiHa and C4-I cells represent biologically distinct models of cervical cancer [85, 86]. Both are human cervical squamous cell carcinoma cell lines derived from primary tumours containing integrated HPV genomes, with SiHa harbouring HPV16 and C4-I harbouring HPV18. We have previously shown that HPV16 E6 transcription is relatively low in SiHa cells, whereas telomerase expression is constitutively high due to the activating C250T mutation in the TERT promoter [87]. In contrast, C4-I cells express HPV18 E6 mRNA at levels more than ten-fold higher than HPV16 E6 in SiHa cells, while exhibiting lower telomerase expression [87]. These biological differences may explain why CPP-pep11 displayed comparable overall efficacy in the two cell lines despite the weaker interaction with HPV18 E6 predicted by AlphaFold2. Collectively, these findings suggest that the biological activity of CPP-pep11 is influenced not only by its predicted binding affinity for E6 but also by the extent to which tumour cells rely on E6-mediated pathways for their growth and survival.

Colony formation assays demonstrated that CPP-pep11 is able to reduce clonogenic potential, decreasing both the number and size of colonies in a concentration-dependent manner. These results indicate that CPP-pep11 impairs long-term cell survival and proliferative capacity in HPV-positive cancer cells, consistent with restoration of p53 function. The subcellular localization of E6 is critical for its various functions, including interactions with p53 and other cellular proteins, and may influence downstream pathways such as apoptosis and cell growth [88]. Notably, CPP-pep11 was primarily detected in the cytoplasm as discrete cytoplasmic puncta indicating that the peptide is largely retained in endocytic vesicles without being degraded consistent with previous reports that the interaction between E6 and E6AP predominantly occurs in this compartment [89]. This is particularly relevant because p53 degradation takes place mainly in the perinuclear cytoplasmic region, where the trimeric complex E6-E6AP-p53 is concentrated [90, 91], and E6 also facilitates the nuclear export of p53 for proteasomal degradation [92, 93]. The targeting of E6-mediated p53 degradation has gained increasing attention as a strategy to restore physiological p53 protein levels [94–96]. Importantly, beyond the innovation of CPP conjugated molecule, in our research, the use of a peptide interfering with HPV E6-mediate degradation of p53 directly, led to a marked increase in p53 levels particularly evident after 72 h of CPP-pep11 treatment. In contrast, the scrambled control CPP-SCRpep11 displayed poor cytoplasmic distribution and accumulated mainly in insoluble fractions, likely due to altered amphipathic properties and reduced solubility. The Biot-CPP-OVA₂₅₇₋₂₆₄ control peptide was unable to cross the cell membrane showing negligible cellular uptake. These results highlight the importance of both HPV E6 sequence specificity and CPP conjugation for efficient cellular delivery [97, 98].

Similarly, other studies demonstrated the effectiveness of small molecules capable of inhibiting p53 degradation, either directly or through interaction with the HPV E6 oncoprotein. One example is the small molecule Reactivation of p53 and Induction of Tumour Apoptosis (RITA), that was shown to inhibit the binding of p53 to E6AP thus impairing its ubiquitination. In cervical cancer models, RITA suppressed cancer cell proliferation and induced apoptosis, an effect associated with increased levels of p53 [99]. Likewise, the bacterial azurin derived peptide p28 (Leu50-Asp77) has been reported to exert anti-proliferative effects in breast cancer cells by reactivating p53 through the reduction of its degradation [100]. However, p28 has no effect in cancers with null-p53 status [101]. Moreover, other studies also showed the efficacy of CPP to improve the transport of molecules inside the cells, as transportan 10 (TP10) that improved the anticancer activity of cisplatin in tumour cells (HeLa, OS143B) and this was attributed to a more effective cellular internalization of the molecule [102].

Consistent with its proposed mechanism of action, CPP-pep11 treatment led to dose- and time-dependent stabilization of p53, as demonstrated by western blotting and immunofluorescence analyses. It is clear that the two cell lines have different p53 localizations; C4-I shows a more cytoplasmic p53 signal than SiHa. However, both show an increase in p53 levels after incubation with the CPP-pep11, with p53 shifting in the nuclear compartment mainly. Notably, increased cytoplasmic and nuclear p53 accumulation correlated with intracellular localization of the peptide, confirming that CPP-pep11 interferes with E6-mediated p53 ubiquitination. The modest variation in p53 levels at the highest peptide concentrations may reflect additional stress responses or saturation of cellular uptake mechanisms. Importantly, these findings support the hypothesis that CPP-pep11 selectively restores p53 activity in HPV-positive cells, providing a mechanistic explanation for the observed anti-proliferative effects.

## Conclusion

Overall, our results demonstrate that CPP-pep11 is a soluble, stable, and efficiently internalized peptide capable of selectively disrupting E6-mediated p53 degradation in HPV-positive cervical cancer cells. The peptide’s design, combining sequence-specific E6 binding with a polyarginine CPP, enabled effective cytoplasmic delivery, nuclear localization, and functional restoration of a key tumour suppressor pathway. These findings provide a strong rationale for further preclinical evaluation of CPP-pep11 as a targeted therapeutic strategy for HPV-driven malignancies and highlight the potential of CPP-conjugated interference peptides as modular tools for intracellular delivery of biologically active sequences.

## Supplementary Information

Supplementary Material 1.

## Data Availability

Data are available at Zenodo (https://zenodo.org/) under the following: https://doi.org/10.5281/zenodo.20441560.

## Abbreviations

Boc: Tert-butyloxycarbonyl
CARA: Computer Aided Resonance Assignment
CPP: Cell-penetrating peptide
DCM: Dichloromethane
DIPEA: N,N-Diisopropylethylamine
DMEM: Dulbecco’s Modified Eagle Medium
DMF: N,N-Dimethylformamide
DMSO: Dimethyl sulfoxide
DPFGSE: Double Pulsed Field Gradient Spin Echo
DQFCOSY: Double Quantum-Filtered Correlated Spectroscopy
DTT: Dithiothreitol
ESI-MS: Electrospray ionization mass spectrometry
FBS: Foetal bovine serum
Fmoc: 9-fluorenylmethoxycarbonyl
HBTU: 2-(1H-benzotriazol-1-yl)-1,1,3,3-tetramethyluronium hexafluorophosphate
HF: Hydrofluoric acid
HPVs: Human papillomaviruses
MBHA: 4-Methylbenzhydrylamine
NOEs: Nuclear Overhauser Effects
NOESY: Nuclear Overhauser Enhancement Spectroscopy
OVA: Ovalbumin
PFA: Paraformaldehyde
RITA: Reactivation of p53 and Induction of Tumour Apoptosis
ROESY: Rotating Frame Overhauser Enhancement Spectroscopy
RP-HPLC: Reverse-phase high-performance liquid chromatography
RTCA: Real-time cell analysis
TFA: Trifluoroacetic acid
TIS: Triisopropylsilane
TOCSY: Total Correlation Spectroscopy
TSP: Trimethylsilyl-3-propionic acid sodium salt
