# Supplementary material for: Development of a cell-penetrating peptide for intracellular delivery of a biologically active HPV E6 inhibitor in cervical cancer cells
Source: J Exp Clin Cancer Res. 2026 Aug 12;45:233. doi: 10.1186/s13046-026-03805-4 (PMC13746660; doi:10.1186/s13046-026-03805-4)
Supplement: Supplementary file 1 [file 13046_2026_3805_MOESM1_ESM.docx]

Supplementary materials

**Development of a cell-penetrating peptide for intracellular delivery of a biologically active HPV E6 inhibitor in cervical cancer cells**

Luisa Dassi^1,^ ^†^, Anna Lucia Tornesello^2,^ ^†,*^, Marian Vincenzi^3^, Marilisa Leone^3^, Vincenzo Ingangi^4^, Wuyuan Lu ^5^, Andrea Cerasuolo^1^, Tiziana Pecchillo Cimmino^1^, Sara Amiranda^1^, Mariapia Napolitano^1^, Palma Tirino^1^, Franca Maria Tuccillo^1^, Luigi Buonaguro^2^, Vincenza De Gregorio^6^, Giorgia Imparato^7^, Franco Maria Buonaguro^1^, Maria Lina Tornesello^1*^

^1^Molecular Biology and Viral Oncology Unit, Istituto Nazionale Tumori IRCCS Fondazione G. Pascale, Napoli, Italy

^2^Innovative Immunological Models Unit, Istituto Nazionale Tumori IRCCS Fondazione G. Pascale, Napoli, Italy

^3^Institute of Biostructures and Bioimaging, National Research Council of Italy (IBB-CNR), Napoli, Italy

^4^Preclinical Models of Tumor Progression Unit, Istituto Nazionale Tumori IRCCS Fondazione G. Pascale, Napoli, Italy

^5^Shanghai Institute of Infectious Disease and Biosecurity, School of Basic Medical Sciences, Shanghai Medical College, Fudan University, Shanghai, China

^6^Department of Biology University of Naples "Federico II", Via Cinthia, Napoli, Italy

^7^Istituto Italiano Di Tecnologia-IIT, Center for Advanced Biomaterials for Healthcare, Napoli, Italy

^†^These authors have contributed equally to this work

*Correspondence: [a.tornesello@istitutotumori.na.it](mailto:a.tornesello@istitutotumori.na.it); [m.tornesello@istitutotumori.na.it](mailto:m.tornesello@istitutotumori.na.it)

1 **Supplementary Figures and Tables**
  1.1 **Supplementary Figures**

**Figure S1.** Physiochemical properties obtained with Bachem Molecular weight calculator (<https://www.bachem.com/knowledge-center/peptide-calculator/>). Physiochemical properties of: (**A**) pep11 (left) and SCRpep11 (right), (**B**) CPP-pep11 (left) and CPP-SCRpep11 (right), (**C**) Biot-CPP-pep11 (left) and Biot-CPP-SCRpep11 (right), (**D**) CPP-OVA_257-264_ (left), Biot-CPP-OVA_257-264_ (right). The graphs show how the net charge of pep11 and SCRpep11 change dramatically after the addition of CPP sequence (net charge -0.13 versus 5.87). Addition of biotin to the N-terminus does not substantially affect the net charge.

**Figure S2.** Chromatographic profiles and mass spectra of purified peptides. The chromatographic profile of: (**A**) purified CPP-pep11 and (**B**) CPP-SCRpep11 show a main peak at 16.132 min and 18.143 min (gradient from 20 to 40% B in 25 minColumn C18 250x21.2mm), corresponding to peptides with a purity of 95% and 90% respectively. ESI-MS analysis confirm that the experimental m/z-values coincide with the theoretical ones: (**C**) CPP-pep11 experimental [M+3H]^3+^=813.25, theoretical [M+3H]^3+^=812.3; (**D**) CPP-SCRpep11 experimental [M+3H]^3+^=813.25, theoretical [M+3H]^3+^=812.3. The chromatographic profile of purified Biot-CPP-pep11 (**E**) and Biot-CPP-SCRpep11 (**F**) shows a main peak at 18.164 min and 18.167 min respectively. The purity of both peptides is above 95%. ESI-MS analysis confirm that the experimental m/z-values coincide with the theoretical ones: (**G**) Biot-CPP-pep11 experimental [M+3H]^3+^=888.43, theoretical [M+3H]^3+^=887.6; (**H**) Biot-CPP-SCRpep11 experimental [M+3H]^3+^=888.62, theoretical [M+3H]^3+^=887.6. The chromatographic profiles of (**I**) purified CPP-OVA_257-264_ and purified Biot-CPP-OVA_257-264_ (**M**) show a main peak at 2.851 min (purity: 80%) and 3.313 min (purity 85%) respectively (gradient from 20 to 80% B in 15min, column C18 150x4.6mm). LC-MS analysis (LC-TOF 1200 Agilent) confirm that the experimental m/z-values coincide with the theoretical ones: (**L**) CPP-OVA_257-264_ experimental [M+3H]^3+^=634.39, theoretical [M+3H]^3+^=633.4; (**N**) Biot-CPP-OVA_257-264_ experimental [M+3H]^3+^=709.74, theoretical [M+3H]^3+^=703.5.

**Figure S3.** Chromatographic profiles and mass spectra of CPP-pep11 after 72h of reaction in oxidation condition. (**A**) Chromatographic profiles of CPP-pep11 after 72h DMSO mediated oxidation and CPP-pep11 at T= 0 (gradient from 20 to 40% B in 35 min). The chromatogram shows no change in retention time and mass spectrum (**B**) confirmed the absence of both intermolecular and intramolecular disulfide bond formation.

**Figure S4.**NMR investigation of the CPP-pep11 peptide (1.13 mM concentration) in H_2_O/D_2_O 90/10 v/v with 8 mM DTT-d10; pH=3.2. (**A**) The 1D [^1^H] NMR spectrum. (**B**) Comparison of the amide/side chain proton correlation regions of the 2D [^1^H, ^1^H] TOCSY (left) and NOESY (right) spectra. C) Aromatic - amide proton correlation region of the 2D [^1^H, ^1^H] NOESY spectrum.

**Figure S5.**NMR investigation of the Biot-CPP-pep11 (770 μM concentration) peptide in H_2_O/D_2_O 90/10 v/v with 8 mM DTT-d10; pH=4.97. (**A**) The 1D [^1^H] NMR spectrum. (**B**) Comparison of the amide/side chain proton correlation regions of the 2D [^1^H, ^1^H] TOCSY (left) and NOESY (right) spectra. (**C**) Aromatic - amide proton correlation region of the 2D [^1^H, ^1^H] NOESY spectrum.

**Figure S6.**NMR investigation of the CPP-SCRpep11 peptide (1 mM concentration) in H_2_O/D_2_O 90/10 v/v with DTT-d10 (8 mM concentration); pH=3.9. (**A**) The 1D [^1^H] NMR spectrum; pH=3.6. (**B**) Comparison of the amide/side chain proton correlation regions of the 2D [^1^H, ^1^H] TOCSY (left) and NOESY (right) spectra. (**C**) Aromatic - amide proton correlation region of the 2D [^1^H, ^1^H] NOESY spectrum.

**Figure S7.**NMR investigation of the Biot-CPP-SCRpep11 peptide (970 μM concentration) in H_2_O/D_2_O 90/10 v/v with DTT-d10 (8 mM concentration); pH=3.6. (**A**) The 1D [^1^H] NMR spectrum. (**B**) Comparison of the amide/side chain proton correlation regions of the 2D [^1^H, ^1^H] TOCSY (left) and NOESY (right) spectra. (**C**) Aromatic - amide proton correlation region of the 2D [^1^H, ^1^H] NOESY spectrum.

**Figure S8.** Results of secondary structure prediction for the CPP-pep11 and CPP-SCRpep11 peptides by the JPred4 webserver. JPred4 [1] relies on the Jnet algorithm and includes “Lupas” that performs predictions of coiled coils regions with windows equal to 14, 21, 28; “Jnetconf” refers to the confidence of the prediction: the higher is better. “Jnetsol” predicts residues with low solvent accessibility with 25,5,0 indicating buried residues with less than 25%, 5% and 0% solvent accessibility, respectively) and “B” stands for buried.

**Figure S9**. Insights into peptide secondary structure by chemical shift analysis [2, 3]. Evaluation of chemical shift deviations ΔΔHα=(δHα_obs_ - δHα_rc_) between observed Hα chemical shift for diverse “pep11” peptide variants (δHα_obs_) and corresponding random coil values (δHα_rc_). Random coil chemical shifts were estimated considering T=298K and the following pH values: 4.97 for Biot-CPP-pep11; 3.2 for CPP-pep11; 3.6 for Biot-CPP-SCRpep11; 3.9 for CPP-SCRpep11. Data are missing for the first six arginine residues due to ambiguous resonance assignments.

**Figure S10.** Schematic representation of short (intra or sequential) and medium range (defined as 2(i, i+2)-5(i, i+5) residues apart) NOEs (=Nuclear Overhauser Effects), characteristic of diverse secondary structure elements [4], for the different “pep11” variants. "dxy(i, i+1)" represents a NOE correlation involving two protons (i.e., x and y) positioned within the i and i+1 amino acid residues, respectively. Each bar is provided with a thickness that is proportional to the corresponding NOE intensity. The graph was generated with CYANA (v. 2.1).

**Figure S11. Comparison between HPV16 E6 and HPV18 E6 proteins.** Protein domain organization (top) and primary sequence (bottom) of E6 from HPV16 (Uniprot [5] entry P03126) (**A**) and HPV18 (Uniprot [5] entry P06463) (**B**). Zn^2+^ coordinating cysteines are highlighted in green and underlined on the E6 amino acid sequences; additional cysteines are indicated by the yellow marks. E6N and E6C stand for N-and C-terminal Zinc binding domains, respectively. (**C**) Sequence identity assessment between HPV16 E6 and HPV18 E6. The alignment and percentage of identity represent outputs from the SIM – Alignment Tool [6] for Protein Sequences in EXPASY [7], <https://web.expasy.org/sim/>, access date 20 April 2026]. The residues belonging to the E6N and E6C regions are included in the red and blue boxes, respectively, and those from the α-helix linker between E6N and E6C are highlighted within the green box. “*” indicates identical residues within the two sequences. To be consistent with previous published reference structure data [8], that were employed as well for further analyses and comparison purposes, the starting sequence numbers were changed from “1” (i.e., Uniprot sequences) to “-6” for HPV16 E6 and “-1” for HPV18 E6.

**Figure S12. Comparison between the cryo-EM structure of the E6AP/HPV16 E6/p53 complex and the AF2 best model of HPV16 E6/CPP-pep11 peptide complex.** (**A, B**) Ribbon representation of the HPV16 E6 structure (i.e., chain C from the EM structure of the human E6AP/HPV16 E6/p53 complex, PDB entry code 9CHT [8]) in complex with the HPV16 E6 binding domain of E6AP (named E6APpep, residue range I401-R418 from the chain A of the PDB entry code 9CHT). Other residues of the PDB entry are not shown to simplify structure visualization. The E6N, α-helix linker and E6C regions of HPV16 E6 are coloured yellow, light green and cyan, respectively. The E6APpep is reported in magenta. Two different orientations rotated by 90 degrees are shown in A and B panels, respectively. (**C, D**) The best AF2 [9, 10] model of HPV16 E6 in complex with the CPP-pep11 peptide. The E6N, α-helix linker and E6C regions of HPV16 E6 are highlighted in red, dark green and blue, respectively. The CPP-pep11 peptide (sequence: 1-RRRRRR-EFGSGCSCIVCIGLI-21) is shown in black. Two different orientations (90 degrees rotation) of the complex are featured in C and D panels, respectively.

**Figure S13. AF2 modelling of the HPV16 E6/CPP-pep11 complex.** (**A**) Ribbon representation of the 2^nd^ best AF2 [9, 10] model obtained for the HPV16 E6/CPP-pep11 complex. Red, dark green and blue colours are, respectively, used to highlight the E6N, α-helix linker and E6C regions of HPV16 E6 whereas, black colour is employed for the CPP-pep11 peptide (sequence: RRRRRR-EFGSGCSCIVCIGLI). The surface of the HPV16 E6 is shown as well. (**B**) 2D diagram of intermolecular interactions generated with LigPlot+ [11, 12] for the HPV16 E6/CPP-pep11 complex shown in (**A**). Carbon, nitrogen, and oxygen atoms are indicated by black, blue, and red spheres, respectively. A maximum cutoff for H-acceptor distance of 2.7 Å and donor-acceptor of 3.35 Å were set to identify H-bonds (See light green lines). The yellow line designates the intermolecular disulfide bridge between C51 (HPV16 E6) and C17 (CPP-pep11). Disulfide bridges are assigned by LigPlot+ considering a cutoff distance between two sulfur atoms equal to 3 Å [11-13], <https://www.ebi.ac.uk/thornton-srv/software/HBPLUS/manual.html>]. HPV16 E6 and CPP-pep11 residues involved in non-bonded interactions are labeled with the one-letter amino acid code and residue number and represented by red and pink crescents with bristles, respectively.

**Figure S14. AF2 modelling of the HPV16 E6/CPP-pep11 complex.** (**A**) Ribbon representation of the 3^rd^ best AF2 [9, 10] model obtained for the HPV16 E6/CPP-pep11 complex. The red, dark green and blue colours are, respectively, used to represent the E6N, α-helix linker and E6C regions of HPV16 E6 whereas, the CPP-pep11 peptide is coloured black. The surface of HPV16 E6 is shown as well. (**B**) 2D diagram of intermolecular interactions produced with LigPlot+ [11, 12] for the HPV16 E6/CPP-pep11 complex shown in (**A**). Carbon, nitrogen, and oxygen atoms are represented by black, blue, and red spheres, respectively. H-bonds are represented by solid light green lines. To detect H-bonds the following maximum distance cutoffs were considered: 2.7 Å (H-acceptor) and 3.35 Å (donor-acceptor). Red and pink crescents with bristles indicate, respectively, HPV16 E6 and CPP-pep11 residues (labeled with the one-letter amino acid code and sequence numbers) involved in non-bonded interactions.

**Figure S15. AF2 modelling of the HPV16 E6/CPP-pep11 complex.** (**A**) Ribbon representation of the 4^th^ best AF2 [9, 10] model obtained for the HPV16 E6/CPP-pep11 complex. Red, dark green and blue colours are, respectively, used to indicate the E6N, α-helix linker and E6C regions of HPV16 E6 whereas. the CPP-pep11 peptide (sequence: RRRRRR-EFGSGCSCIVCIGLI) is reported in black. The surface of the HPV16 E6 is shown as well. (**B**) 2D diagram of intermolecular interactions generated with LigPlot+ [11, 12] for the HPV16 E6/CPP-pep11 complex shown in (**A**). Carbon, nitrogen, and oxygen atoms are indicated by black, blue, and red spheres, respectively. Intermolecular H-bonds are highlighted by solid light green lines. A maximum cutoff for H-acceptor distances equal to 2.7 Å and donor-acceptor equal to 3.35 Å were set to find H-bonds. The intermolecular disulfide bridge between C51 (HPV16 E6) and C17 (CPP-pep11) is highlighted by a solid yellow line. Disulfide bridges are assigned by LigPlot+ considering a cutoff distance between two sulfur atoms equal to 3 Å [11-13], <https://www.ebi.ac.uk/thornton-srv/software/HBPLUS/manual.html>]. HPV16 E6 and CPP-pep11 residues involved in further non-bonded interactions are labeled with the one-letter amino acid code and residue number and represented by red and pink crescents with bristles, respectively.

**Figure S16. AF2 modelling of the HPV16 E6/CPP-pep11 complex.** (**A**) Ribbon representation of the 5^th^ best AF2 [9, 10] model obtained for the HPV16 E6/CPP-pep11 complex. Red, dark green and blue are, respectively, used to indicate E6N, α-helix linker and E6C regions of HPV16 E6 structure whereas Black is used to indicate the CPP-pep11 peptide (sequence: RRRRRR-EFGSGCSCIVCIGLI). The surface of the HPV16 E6 is shown as well. (**B**) 2D diagram of intermolecular interactions generated with LigPlot+ [11, 12] for the HPV16 E6/CPP-pep11 complex shown in (**A**). Carbon, nitrogen, and oxygen atoms are indicated by black, blue, and red spheres, respectively. A maximum cutoff for H-acceptor distances of 2.7 Å and donor-acceptor distances of 3.35 Å were set to identify H-bonds (indicated by light green solid lines). The possible intermolecular disulfide bridge between C51 (HPV16 E6) and C17 (CPP-pep11) is highlighted by a solid yellow line. Disulfide bridges are assigned by LigPlot+ considering a cutoff distance between two sulfur atoms equal to 3 Å [11-13], <https://www.ebi.ac.uk/thornton-srv/software/HBPLUS/manual.html>]. HPV16 E6 and CPP-pep11 residues involved in non-bonded interactions are labeled with the one-letter amino acid code and residue number and represented by red and pink crescents with bristles, respectively.

**Figure S17. AF2 predicted models of the HPV16 E6/CPP-pep11 peptide complex and RMSD analysis** (**A**) The backbone atoms (i.e., N, Cα, C’, O) of the 2^nd^ to 5^th^ predicted AF2 structures of the HPV16 E6 in complex with CPP-pep11 peptide are overlayed with the corresponding ones in the best model. The E6N, α-helix linker and E6C regions of HPV16 E6 are coloured red, dark green and blue, respectively, whereas, the CPP-pep11 peptide is shown in black. (**B**) The RMSD values reported in each table refer to pairwise RMSD evaluation following superposition on backbone atoms of all possible combinations of the best five AF2 predictions.

**Figure S18. Comparison between the Cryo-EM structure of E6AP/HPV16 E6/p53 complex and AF2 best model of HPV18 E6/CPP-pep11 peptide complex.** (**A, B**) Ribbon representation of the HPV16 E6 structure (i.e., chain C from the Cryo-EM coordinates with PDB entry code 9CHT [8]) in complex with the HPV16 E6 binding domain of E6AP (named E6APpep, residue range I401-R418 from the chain A of the PDB entry 9CHT [8]). Other E6 residues included in the PDB entry are not shown to simplify structure visualization. The yellow, light green and cyan colours are employed, respectively, to highlight the E6N, α-helix linker and E6C regions of HPV18 E6. The E6APpep is colored magenta. Two different views of the E6AP peptide/HPV16 E6 protein complex rotated by 90 degrees are featured in the A and B panels, respectively. (**C, D**) The best AF2 [9, 10] model of the HPV18 E6 in complex with CPP-pep11 peptide. Red, dark green and blue are, respectively, used to indicate E6N, α-helix linker and E6C regions. CPP-pep11 (sequence: RRRRRR-EFGSGCSCIVCIGLI) is shown in black. The C and D panels present two different orientations of the AF2 prediction for the HPV18 E6 protein/CPP-pep11 peptide complex.

**Figure S19. AF2 modelling of the HPV18 E6/CPP-pep11 complex.** (**A**) Ribbon representation of the 2^nd^ best AF2 [9, 10] model obtained for the HPV18 E6/CPP-pep11 complex. Red, dark green and blue colours indicate, respectively, the E6N, α-helix linker and E6C regions of HPV18 E6 whereas, the CPP-pep11 peptide (sequence: RRRRRR-EFGSGCSCIVCIGLI) is reported in black. The surface of the HPV18 E6 is shown as well. (**B**) 2D diagram of intermolecular interactions generated with LigPlot+ [11, 12] for the HPV18 E6/CPP-pep11 complex presented in (**A**). Carbon, nitrogen, and oxygen atoms are indicated by black, blue, and red spheres, respectively. H-bonds are designated with solid light green lines. Maximum cutoffs for H-acceptor distances of 2.7 Å and donor-acceptor of 3.35 Å were set to find H-bonds. HPV18 E6 and CPP-pep11 residues involved in non-bonded interactions are labeled with the one-letter amino acid code and residue number and represented by red and pink crescents with bristles, respectively.

**Figure S20. AF2 modelling of the HPV18 E6/CPP-pep11 complex.** (**A**) Ribbon representation of the 3^rd^ best AF2 [9, 10] model obtained for the HPV18 E6/CPP-pep11 complex. Red, dark green and blue are, respectively, used for the E6N, α-helix linker and E6C regions of HPV18 E6 structure whereas, the black colour refers to the CPP-pep11 peptide (sequence: RRRRRR-EFGSGCSCIVCIGLI). The surface of the HPV18 E6 is shown as well. (**B**) 2D diagram of intermolecular interactions generated with LigPlot+ [11, 12] for the HPV18 E6/CPP-pep11 complex shown in (**A**). Carbon, nitrogen, and oxygen atoms are indicated by black, blue, and red spheres, respectively. H-bonds are designated with solid light green lines. Maximum cutoffs for H-acceptor distances of 2.7 Å and donor-acceptor of 3.35 Å were set to find H-bonds. HPV18 E6 and CPP-pep11 residues involved in non-bonded interactions are labeled with the one-letter amino acid code and residue number and represented by red and pink crescents with bristles, respectively.

**Figure S21. AF2 modelling of the HPV18 E6/CPP-pep11 complex.** (**A**) Ribbon representation of the 4^th^ best AF2 [9, 10] model obtained for the HPV18 E6/CPP-pep11 complex. Red, dark green and blue colours are, respectively, employed to highlight the E6N, α-helix linker and E6C regions of HPV18 E6 whereas, the CPP-pep11 peptide (sequence: RRRRRR-EFGSGCSCIVCIGLI) is reported in black. The surface of the HPV18 E6 is shown as well. (**B**) 2D diagram of intermolecular interactions generated with LigPlot+ [11, 12] for the HPV18 E6/CPP-pep11 complex shown in (**A**). Carbon, nitrogen, and oxygen atoms are represented by black, blue, and red spheres, respectively. H-bonds are indicated with solid light green lines. Maximum cutoffs for H-acceptor distances of 2.7 Å and donor-acceptor of 3.35 Å were set to detect H-bonds. HPV18 E6 and CPP-pep11 residues involved in non-bonded interactions are labeled with the one-letter amino acid code and residue number and represented by red and pink crescents with bristles, respectively.

**Figure S22. AF2 modelling of the HPV18 E6/CPP-pep11 complex.** (**A**) Ribbon representation of the 5^th^ best AF2 [9, 10] model obtained for the HPV18 E6/CPP-pep11 complex. The red, dark green and blue colours are, respectively, employed to highlight the E6N, α-helix linker and E6C regions of HPV18 E6 whereas, the CPP-pep11 peptide (sequence: RRRRRR-EFGSGCSCIVCIGLI) is coloured black. The surface of the HPV18 E6 is shown as well. (**B**) 2D diagram of intermolecular interactions generated with LigPlot+ [11, 12] for the HPV18 E6/CPP-pep11 complex shown in (**A**). Carbon, nitrogen, and oxygen atoms are indicated by black, blue, and red spheres, respectively. H-bonds are represented by solid light green lines. Maximum cutoffs for H-acceptor distances of 2.7 Å and donor-acceptor of 3.35 Å were set to detect H-bonds. HPV18 E6 and CPP-pep11 residues involved in further non-bonded interactions are labeled with the one-letter amino acid code and residue number and represented by red and pink crescents with bristles, respectively.

**Figure S23. AF2 models obtained for HPV18 E6/CPP-pep11 peptide complex and RMSD analysis.** (**A**) The backbone atoms (i.e., N, Cα, C’, O) of the 2^nd^ to 5^th^ predicted AF2 structures of the HPV18 E6 in complex with CPP-pep11 peptide are superimposed with the corresponding ones in the best model. The E6N, α-helix linker and E6C regions of HPV16 E6 are coloured red, dark green and blue, respectively, whereas, the CPP-pep11 peptide is shown in black. (**B**) The RMSD values reported in each table refer to pairwise RMSD evaluation following superposition on backbone atoms of all possible combinations of the best five AF2 predictions.

**Figure S24. Imaging of CPP-pep11 in C4-I cells.** Representative images of C4-I cells incubated with Biot-CPP-pep11 (0, 10, 20 μM). Cells were then fixed and stained for the peptide (green), α-tubulin (red), and nuclei (blue). Scale bar: 10 μm.

**Figure S25. Nuclear localization of Biot-CPP-pep11 (20 µM) in SiHa.** Representative image of SiHa cells incubated for 48 h with 20 µM Biot-CPP-pep11 (green). CPP-pep11 (green) displays both cytoplasmic distribution and marked nuclear localization. Scale bar: 10 µm.

**Figure S26. Cellular localization of Biot-CPP-SCRpep11 and Biot-CPP-OVA_257-264_ in HPV16-positive SiHa cells.** Representative immunofluorescence images of HPV16-positive SiHa cells untreated (control) or treated for 48 h with increasing concentrations (0.5, 2, 5, 10 μM) of Biot-CPP-SCRpep11 (**A**) and Biot-CPP-OVA_257-264_ (**B**). Cells were stained for Biot-CPP-SCRpep11 or Biot-CPP-OVA_257-264_ (green), α-tubulin (red), and nuclei (blue). Images acquired at 48 h show the Biot-CPP-SCRpep11 to aggregate at greater concentrations and persist outside the cells thus limiting its cellular uptake. No evident signal is observed in cells treated with different concentrations of Biot- CPP-OVA_257-264_ (0.5, 2, 5, 10 μM). Scale bar: 10 µm.

**Figure S27. Experimental design for Biot-CPP-OVA_257-264_ localization.** SiHa cells were incubated with Biot- CPP-OVA_257_**_-_**_264_ and Streptavidin-AF488 (green signal) for 1h at 37°C, 5% CO_2_. Confocal microscopy analysis shows the peptide to localize around the cell membrane. The post-fixation analysis of cells, after washing and nuclear staining (Hoechst 33342, blue), by confocal microscopy shows a reduction of green signal intensity. Scale bar: 10 µm.

**Figure S28. CPP-pep11 stability over time in cells.** Representative images in confocal microscopy representing Biot-CPP-pep11 (green) at 96h. CPP-pep11 is visible in the cytoplasm maintaining its stability over time. α-tubulin (red), nuclei (blue). Scale bar: 10 μm.

**Figure S29. Basal proliferation kinetics.** Real-time cell proliferation of SiHa (**A**) and C4-I cells (**B**) was assessed by impedance-based measurement. Cell index values were recorded every 15 min for 96 h. Curves show the proliferation kinetics under standard culture conditions. Wells seeded with 2.5 × 10³ cells exhibited a more pronounced exponential growth pattern over time in both cell lines.

**Figure S30. Real-time monitoring of cell proliferation in SiHa cells by cell index assay.** Cells were seeded in E-plates and allowed to adhere for 24 h before treatment with CPP-SCRpep11 at the indicated concentrations (0, 0.5, 2, 5, 10, and 20 µM). Cell proliferation was monitored using the xCELLigence RTCA System, and impedance values (expressed as cell index) were recorded every 15 min for 96 h. (**A**) Cell index values were normalized to the time point immediately after serum addition. (**B**) Relative growth was calculated and expressed as fold change compared to untreated controls. Data are presented as mean ± mean ± SD of two independent experiments performed in quadruplicate.

**Figure S31. Real-time monitoring of cell proliferation in C4-I cells by cell index assay.** Cells were seeded in E-plates and allowed to adhere for 24 h before treatment with CPP-SCRpep11 at the indicated concentrations (0, 0.5, 2, 5, 10, and 20 µM). Cell proliferation was monitored using the xCELLigence RTCA System, and impedance values (expressed as cell index) were recorded every 15 min for 96 h. (**A**) Cell index values were normalized to the time point immediately after serum addition. (**B**) Relative growth was calculated and expressed as fold change compared to untreated controls. Data are presented as mean ± mean ± SD of two independent experiments performed in quadruplicate.

**Figure S32. Assessment of cell viability after CPP peptides treatment over time** (**A**) Schematic representation of the experimental design. Counting of the cells was performed using the trypan blue assay. Cells were plated and allowed to adhere 24h prior to CPP-pep11 and CPP-SCRpep11 incubation for 6h. Cells at T0h show the starting point. Treated cells were counted for living cells after 24, 48, and 72h from the CPP incubation. (**B-C**) Graphs show the results for SiHa cells representing the number of cells/mL at each time point for each concentration used respectively for CPP-pep11 and CPP-SCRpep11. Cell viability is strongly reduced in cells treated with 20µM CPP-pep11 at 72h and a significant decrease in cell viability is observable at 5 and 10 µM CPP-SCRpep11 after 24h. (**D-E**) Graphs show the results for C4-I cell line where the number of cells/mL at each time point for each concentration used of CPP-pep11 or CPP-SCRpep11. Cell viability is strongly reduced in cells treated with 20µM CPP-pep11 at 72h and a significant decrease in cell proliferative activity is observable over time at 5 and 10 µM CPP-SCRpep11. Experiments were performed in duplicate at least 3 times.

**1.2 Supplementary Tables**

**Table S1.** ^1^H Chemical shifts for Biot-CPP-pep11 (770 µM concentration) in H_2_O/D_2_O 90/10 v/v pH 4.97. Ambiguous assignments are reported in red. Unambiguous chemical shifts assignments could not be achieved for the arginine residues n.2,3,4,5 and 6 due to spectral overlaps. However, ^1^H_N_ signals from Arginine’s are gathered between 8.38 and 8.45 ppm.

| **Residue** | **H_N_** | **Hα** | **Hβ** | **Hγ** | **Others** |
| --- | --- | --- | --- | --- | --- |
| **Biot** |  |  |  |  | (H25,26) 2.77, 2.99  (H27) 4.62  (H29 4.40  (H28) 3.32  (H23,24) 2.34  (H21,22) 1.66,1.55  (H17,18) 1.38  (H19,20) 1.55  @See below for the employed biotinoyl ^1^H nomenclature |
| **1 R** | 8.34 | 4.27 | 1.75 | 1.72 | Hδ  Hε |
| **2 R** |  |  |  |  | Hδ  Hε |
| **3 R** |  |  |  |  | Hδ  Hε |
| **4 R** |  |  |  |  | Hδ  Hε |
| **5 R** |  |  |  |  | Hδ  Hε |
| **6 R** |  |  |  |  | Hδ  Hε |
| **7 E** | 8.45 | 4.31 | 1.84-1.96 | 2.23 |  |
| **8 F** | 8.34 | 4.62 | 2.98-3.18 |  | Hδ 7.28  Hε 7.33  Hζ 7.31 |
| **9 G** | 8.38 | 3.92-3.99 |  |  |  |
| **10 S** | 8.34 | 4.47 | 3.90 |  |  |
| **11 G** | 8.59 | 4.02 |  |  |  |
| **12 C** | 8.22 | 4.58 | 2.95 |  |  |
| **13 S** | 8.48 | 4.52 | 3.89 |  |  |
| **14 C** | 8.34 | 4.55 | 2.93 |  |  |
| **15 I** | 8.22 | 4.18 | 1.86 | 1.19-1.51 | δCH3 0.90 |
| **16 V** | 8.24 | 4.15 | 2.05 | 0.92-0.93 |  |
| **17 C** | 8.48 | 4.53 | 2.89 |  |  |
| **18 I** | 8.31 | 4.19 | 1.87 | 1.19-1.51 | δCH3 0.91 |
| **19 G** | 8.44 | 3.94 |  |  |  |
| **20 L** | 8.08 | 4.38 | 1.63 | 1.63 | δCH3 0.88-0.93 |
| **21 I** | 8.08 | 4.16 | 1.86 | 1.19-1.51 | 0.92  CONH_2_ 7.11-7.63 |

^@^

Atom numbering in the biotinoyl chain follows that reported within the BMRB (Biological Magnetic Resonance databank (<https://bmrb.io/metabolomics/mol_summary/show_data.php?id=bmse000227>, access date 16/03/2026)).

| **Residue** | **H_N_** | **Hα** | **Hβ** | **Hγ** | **Others** |
| --- | --- | --- | --- | --- | --- |
| **1 R** |  | 4.05 | 1.91 | 1.64 | Hδ 3.21 |
| **2 R** | 8.81 | 4.38 | 1.79 | 1.66 | Hδ 3.19  Hε 7.22 |
| **3 R** | 8.607 | 4.344 | 1.77 | 1.66 | Hδ 3.19  Hε 7.19 |
| **4 R** | 8.515 | 4.33 | 1.75 | 1.64 | Hδ 3.19  Hε 7.19 |
| **5 R** | 8.46 | 4.29 | 1.77 | 1.61 | Hδ 3.20  Hε 7.19 |
| **6 R** | 8.44 | 4.27 | 1.74 | 1.58 | Hδ 3.17  Hε 7.14 |
| **7 E** | 8.41 | 4.30 | 1.86-1.96 | 2.24 |  |
| **8 F** | 8.34 | 4.64 | 3.03-3.17 |  | Hδ 7.26  Hε 7.34  Hζ 7.30 |
| **9 G** | 8.38 | 3.92-4.02 |  |  |  |
| **10 S** | 8.33 | 4.48 | 3.90 |  |  |
| **11 G** | 8.58 | 4.01 |  |  |  |
| **12 C** | 8.21 | 4.57 | 2.94 |  |  |
| **13 S** | 8.48 | 4.50 | 3.90 |  |  |
| **14 C** | 8.33 | 4.55 | 2.92 |  |  |
| **15 I** | 8.21 | 4.16 | 1.84 | 1.47-1.19 | γCH3 0.89  δCH3 0.88 |
| **16 V** | 8.22 | 4.15 | 2.03 | 0.90-0.92 |  |
| **17 C** | 8.48 | 4.51 | 2.88 |  |  |
| **18 I** | 8.30 | 4.18 | 1.88 | 1.47-1.19 | γCH3 0.92  δCH3 0.92 |
| **19 G** | 8.43 | 3.93 |  |  |  |
| **20 L** | 8.07 | 4.37 | 1.61 | 1.61 | 0.92-0.87 |
| **21 I** | 8.07 | 4.14 | 1.87 | 1.47-1.19 | γCH3 0.92  δCH3 0.92  CONH_2_ 7.09-7.62 |

**Table S2.** ^1^H Chemical shifts for CPP-pep11 (1.13 mM concentration) in H_2_O/D_2_O 90/10 v/v pH 3.2.

**Table S3.** ^1^H Chemical shifts Biot-SCRpep11 (970 µM) in H_2_O/D_2_O 90/10 v/v pH 3.6. The assignments of arginine residues 3-6 within the CPP moiety are missing due to spectral overlaps. Other ambiguous assignments are highlighted in red.

| **Residue** | **H_N_** | **Hα** | **Hβ** | **Hγ** | **Others** |
| --- | --- | --- | --- | --- | --- |
| **Biot** |  |  |  |  | (H25,26) 2.77, 2.98  (H27) 4.62  (H29) 4.39  (H28) 3.31  (H23,24) 2.33  (H21,22) 1.64,1.56  (H17,18) 1.36  (H19,20) 1.56  @See note in Table S1 for the employed biotinoyl ^1^H nomenclature |
| **1 R** | 8.34 | 4.25 | 1.76 | 1.55 | Hδ 3.18  Hε |
| **2 R** | 8.38 | 4.33 | 1.76 | 1.61 | Hδ 3.17  Hε |
| **3 R** |  |  |  |  | Hδ  Hε |
| **4 R** |  |  |  |  | Hδ  Hε |
| **5 R** |  |  |  |  | Hδ  Hε |
| **6 R** |  |  |  |  | Hδ  Hε |
| **7C** | 8.46 | 4.54 | 2.88 |  |  |
| **8 G** | 8.51 | 3.97 |  |  |  |
| **9 V** | 8.03 | 4.21 | 2.07 | 0.89-0.94 |  |
| **10 S** | 8.45 | 4.53 | 3.86 |  |  |
| **11 I** | 8.28 | 4.22 | 1.88 | 1.45-1.19 | γCH3 0.91  δCH3 0.88 |
| **12 C** | 8.41 | 4.49 | 2.90 |  |  |
| **13 G** | 8.41 | 3.94 |  |  |  |
| **14 L** | 8.04 | 4.37 | 154-1.59 | 1.54/1.59 | 0.87 |
| **15 S** | 8.26 | 4.38 | 3.78 |  |  |
| **16 F** | 8.13 | 4.67 | 3.00-3.12 |  | Hδ 7.20  Hε 7.32  Hζ |
| **17 E** | 8.24 | 4.36 | 1.90-1.99 | 2.27 |  |
| **18 I** | 8.19 | 4.17 | 1.85 | 1.45-1.19 | γCH3 0.91  δCH3 0.88 |
| **19 C** | 8.47 | 4.54 | 2.90 |  |  |
| **20 I** | 8.36 | 4.18 | 1.85 | 1.45-1.19 | γCH3 0.91  δCH3 0.88 |
| **21 G** | 8.47 | 3.90 |  |  | CONH_2_ 7.06-7.43 |

**Table S4.** ^1^H Chemical shifts for CPP-SCRpep11 (1 mM concentration) in H_2_O/D_2_O 90/10 v/v pH=3.9. Ambiguous assignments are reported in red. Due to spectral overlaps it was not possible to get resonance assignments for arginines 4-6.

| **Residue** | **H_N_** | **Hα** | **Hβ** | **Hγ** | **Others** |
| --- | --- | --- | --- | --- | --- |
| **1 R** |  | 4.05 | 1.92 | 1.66 | Hδ 3.22  Hε 7.21 |
| **2 R** | 8.80 | 4.40 | 1.79 | 1.66 | Hδ 3.19  Hε 7.18 |
| **3 R** | 8.61 | 4.35 | 1.77 | 1.69 | Hδ 3.17  Hε 7.18 |
| **4 R** |  |  |  |  | Hδ  Hε |
| **5 R** |  |  |  |  | Hδ  Hε |
| **6 R** |  |  |  |  | Hδ  Hε |
| **7 C** | 8.46 | 4.54 | 2.90 |  |  |
| **8 G** | 8.51 | 3.99 |  |  |  |
| **9 V** | 8.03 | 4.22 | 2.07 | 0.92-0.94 |  |
| **10 S** | 8.44 | 4.54 | 3.86 |  |  |
| **11 I** | 8.28 | 4.24 | 1.91 | 1.21-1.46 | γCH3 0.91  δCH3 0.91 |
| **12 C** | 8.41 | 4.51 | 2.93 |  |  |
| **13 G** | 8.41 | 3.94 |  |  |  |
| **14 L** | 8.04 | 4.38 | 1.62-1.57 | 1.62/1.57 | 0.86-0.91 |
| **15 S** | 8.25 | 4.41 | 3.80 |  |  |
| **16 F** | 8.13 | 4.67 | 3.02-3.13 |  | Hδ 7.21  Hε 7.33  Hζ 7.31 |
| **17 E** | 8.24 | 4.38 | 1.91-1.99 | 2.29 |  |
| **18 I** | 8.18 | 4.18 | 1.87 | 1.21-1.46 | γCH3 0.91  δCH3 0.91 |
| **19 C** | 8.47 | 4.55 | 2.92 |  |  |
| **20 I** | 8.35 | 4.18 | 1.88 | 1.21-1.46 | γCH3 0.91  δCH3 0.91 |
| **21 G** | 8.49 | 3.94 |  |  | CONH_2_ 7.08-7.43 |

**Table S5.** List of antibodies used in immunofluorescence and western blotting analyses.

| **Antibody** | **Host** | **Category** | **Clonality** | **Company** | **Dilution** | **Application** |
| --- | --- | --- | --- | --- | --- | --- |
| Anti-α-tubulin | Rabbit | Primary | Monoclonal | Cell signaling, 2125 | 1:200 | IF |
| Phalloidin,  Alexa Fluor 647 | - | - | - | Invitrogen, A22287 | 1:400 | IF |
| Streptavidin,  Alexa Fluor 488 conjugate | - | Secondary | - | Invitrogen, S11223 | 1:200 | IF |
| Anti-rabbit  IgG Alexa Fluor 594 | - | - | - | Abcam, ab150080 | 1:500 | IF |
| Anti-p53 | Mouse | Primary | Monoclonal | Santa cruz, sc-126 | 1:500 | WB |
| Anti-actin | Mouse | Primary | Monoclonal | Merck, MAB1501R | 1:1000 | WB |
| Anti-mouse  IgG-HRP | Goat | Secondary | Polyclonal | Santa cruz, sc-2302 | 1:2000 | WB |
| Streptavidin-HRP conjugate | - | Secondary | - | Invitrogen, SA100-01 | 1:5000 | WB |

**References**

1.	Drozdetskiy A, Cole C, Procter J, Barton GJ: **JPred4: a protein secondary structure prediction server**. *Nucleic Acids Res* 2015, **43**(W1):W389-394.

2.	Wishart DS, Sykes BD, Richards FM: **The chemical shift index: a fast and simple method for the assignment of protein secondary structure through NMR spectroscopy**. *Biochemistry* 1992, **31**(6):1647-1651.

3.	Kjaergaard M, Poulsen FM: **Sequence correction of random coil chemical shifts: correlation between neighbor correction factors and changes in the Ramachandran distribution**. *J Biomol NMR* 2011, **50**(2):157-165.

4.	Wuthrich K: **NMR of Proteins and Nucleic Acids**. New YorK: Wiley; 1986.

5.	**UniProt, Consortium. UniProt: the Universal Protein Knowledgebase in 2025**. *Nucleic Acids Res* 2025, **53**(D1):D609-D617.

6.	Huang X, Miller W: **A Time-Efficient, Linear-Space Local Similarity Algorithm.** . *Adv Appl Math* 1991, **12**(3):337-357.

7.	Wilkins MR, Gasteiger E, Bairoch A, Sanchez JC, Williams KL, Appel RD, Hochstrasser DF: **Protein identification and analysis tools in the ExPASy server**. *Methods Mol Biol* 1999, **112**:531-552.

8.	Kenny S, Iyer S, Gabel CA, Tegenfeldt N, DeMarco AG, Hall MC, Chang L, Davisson VJ, Pol SV, Das C: **Structure of E6AP in complex with HPV16-E6 and p53 reveals a novel ordered domain important for E3 ligase activation**. *Structure* 2025, **33**(3):504-516 e504.

9.	Jumper J, Evans R, Pritzel A, Green T, Figurnov M, Ronneberger O, Tunyasuvunakool K, Bates R, Zidek A, Potapenko A *et al*: **Highly accurate protein structure prediction with AlphaFold**. *Nature* 2021, **596**(7873):583-589.

10.	Varadi M, Anyango S, Deshpande M, Nair S, Natassia C, Yordanova G, Yuan D, Stroe O, Wood G, Laydon A *et al*: **AlphaFold Protein Structure Database: massively expanding the structural coverage of protein-sequence space with high-accuracy models**. *Nucleic Acids Res* 2022, **50**(D1):D439-D444.

11.	Laskowski RA, Swindells MB: **LigPlot+: multiple ligand-protein interaction diagrams for drug discovery**. *J Chem Inf Model* 2011, **51**(10):2778-2786.

12.	Wallace AC, Laskowski RA, Thornton JM: **LIGPLOT: a program to generate schematic diagrams of protein-ligand interactions**. *Protein Eng* 1995, **8**(2):127-134.

13.	**HBPLUS v.3.06 - a hydrogen bond calculation program** [<https://www.ebi.ac.uk/thornton-srv/software/HBPLUS/manual.html>]
